# The role of lipid metabolic reprogramming in tumor microenvironment

**DOI:** 10.7150/thno.82920

**Published:** 2023-03-13

**Authors:** Kai Yang, Xiaokun Wang, Chenghu Song, Zhao He, Ruixin Wang, Yongrui Xu, Guanyu Jiang, Yuan Wan, Jie Mei, Wenjun Mao

**Affiliations:** 1 Department of Thoracic Surgery, The Affiliated Wuxi People's Hospital of Nanjing Medical University, Wuxi, 214023, China.; 2 Department of Oncology, The Affiliated Wuxi People's Hospital of Nanjing Medical University, Nanjing, 210029, China.; 3 The Pq Laboratory of BiomeDx/Rx, Department of Biomedical Engineering, Binghamton University, Binghamton 13850, USA.

**Keywords:** lipid metabolic reprogramming, tumor microenvironment, immunotherapy, therapeutic target

## Abstract

Metabolic reprogramming is one of the most important hallmarks of malignant tumors. Specifically, lipid metabolic reprogramming has marked impacts on cancer progression and therapeutic response by remodeling the tumor microenvironment (TME). In the past few decades, immunotherapy has revolutionized the treatment landscape for advanced cancers. Lipid metabolic reprogramming plays pivotal role in regulating the immune microenvironment and response to cancer immunotherapy. Here, we systematically reviewed the characteristics, mechanism, and role of lipid metabolic reprogramming in tumor and immune cells in the TME, appraised the effects of various cell death modes (specifically ferroptosis) on lipid metabolism, and summarized the antitumor therapies targeting lipid metabolism. Overall, lipid metabolic reprogramming has profound effects on cancer immunotherapy by regulating the immune microenvironment; therefore, targeting lipid metabolic reprogramming may lead to the development of innovative clinical applications including sensitizing immunotherapy.

## Background

The history of drug therapy for malignancies, such as cancer, has developed from chemotherapy to targeted therapy and then to immunotherapy. Currently, immunotherapy is the most promising antitumor treatment 1. Unlike patients on conventional therapies, those who respond to immunotherapy are more likely to achieve high-quality long-term survival with fewer side effects 2. The "immunosurveillance theory" states that the immune system has a complete surveillance function to distinguish and remove "non-self" tumors 3. The core of tumor immunotherapy is to enhance the antitumor immunity by targeting the cancer-immune cycle. This mainly includes the release and presentation of tumor antigens to activate tumor-associated immune cells, and these antigens are delivered and infiltrated into the tumor tissues to enhance their recognition and subsequent elimination 4. However, immune escape and immunosuppression reduce the effectiveness of immunotherapy because of abnormal regulations of the cancer-immune cycle 5. The "tumor immunoediting theory" states that the immune system can not only eliminate tumors but also promote their growth, and this interaction occurs in three stages: clearance, equilibrium, and escape 6. Tumors evade the clearance of the immune system and suppress the antitumor immune response by several mechanisms. Metabolic reprogramming is one of these mechanisms in which the metabolic patterns of tumor and immune cells are altered to meet their developmental requirements and adapt to the complex tumor microenvironment (TME) 7. Therefore, correcting the metabolic reprogramming of cells can help to overcome immune escape and immunosuppression, thereby sensitizing immunotherapy.

Metabolic reprogramming is one of the 14 important hallmarks of tumorigenesis in which the energy metabolism network of tumor cells is reset to support their growth, proliferation, and metastasis under the metabolic stress of the TME and immune surveillance 8. The "starved" tumor cells undergo adaptive metabolic reprogramming including glucose, lipid, and amino acid metabolism 9, 10. Normal cells rely on mitochondrial respiration for energy supply but tumor cells rely mainly on glycolysis even under aerobic conditions; this phenomenon is known as the 'Warburg effect' 11. This effect also modulates cellular lipid metabolism by providing raw materials for lipid synthesis, regulating lipid metabolic signaling pathways, and reducing lipid peroxidation. In addition, key enzymes of the gluconeogenesis pathway are also involved in the regulation of lipid metabolism 12. However, compared with glucose metabolic reprogramming, lipid metabolic reprogramming needs to be further studied.

Lipids are classified as simple and complex lipids; the simple lipids include triglycerides (TAGs), commonly known as fats. The complex lipids include sterols and their esters, phospholipids (PL), and glycolipids. In addition, ketone bodies, although not lipids are important intermediates of fatty acid oxidation (FAO). Lipids play an important function in cellular activities. In addition to serving as a key energy source 13, lipids are the material basis for the synthesis of important biological components 14, involved in intra- and extracellular signal transduction 15, comprise the plasma membrane structure 16, and modify several biomolecules 17. Cells obtain lipids through exogenous uptake and endogenous synthesis for metabolic activities and store excess lipids in lipid droplets (LDs), which finally undergo oxidative decomposition; this whole process is tightly regulated. Lipid metabolism in tumor cells is different from that in normal cells because of genetic mutations, mitochondrial damage, and TME 18. This happens in three stages to meet the high energy demand during growth and to mediate immunosuppression. First, the uptake and de novo synthesis of lipids are enhanced through altered nutritional patterns 19, 20. Then, the synthesized lipids are preferentially allocated to metabolic pathways that contribute to oncogenic modification. Finally, alterations in metabolism have long-term effects on the tumor and immune cells in the TME 21. The key enzymes in lipid metabolism can have abnormal activity and even have new non-metabolic enzyme functions during this process 22.

Lipid metabolic reprogramming not only plays an important role in the oncogenesis and development of tumors but also modifies the TME by affecting the recruitment, survival, and function of immune cells 23. Unlike monoclonal cell populations, tumor cells and the cells of TME interact to form a continuously evolving and heterogenous entity 24. This dynamic change and heterogeneity results in varied lipid metabolism in different types of tumor and immune cells at different stages. Tumor cells in an active proliferative or metastatic state are more dependent on lipid metabolism. Moreover, protumor immune cells also depend on lipid metabolism, whereas antitumor immune cells depend on glucose metabolism 25. In addition, autophagy induced by metabolic stress of the TME 26 and other cell death modes related to lipid peroxidation (including ferroptosis 27, cuproptosis 28, and calcium overload 29) are also associated with cellular lipid metabolism and affect tumor immune responses. Therefore, these modes of cell death are the potential targets for tumor therapy.

Lipid metabolic reprogramming plays a critical role in sensitizing immunotherapy. Immunotherapy combined with therapies targeting lipid metabolism will provide a new direction for tumor treatment 23. Here, we reviewed the characteristics, mechanism, and role of lipid metabolic reprogramming from the perspective of tumor and immune cells in the TME, elaborated on the effects of various cell death modes (especially ferroptosis) on lipid metabolism, and finally summarized the antitumor therapies targeting lipid metabolism to guide the development of new strategies for sensitizing immunotherapy.

## Specific functions of lipids in tumor cells

### Providing energy and material basis

The most prominent function of lipids is to provide energy for cellular activities. TAGs are an important energy supply and storage material for cells. TAGs break down to yield fatty acids (FA), which undergo FAO and mitochondrial oxidative phosphorylation (OXPHOS) to produce large amounts of ATP. Lipids are not preferentially used by cells. Tumor and other immune cells rely on glucose metabolism when the supply is sufficient. However, when cells are activated or starving, especially under the metabolic stress of the TME, they switch to lipids for energy and material basis 30.

In addition, the intermediates of FAO, ketone bodies, are also available for cellular utilization. Immune cells can use ketone bodies as an alternative energy source in the absence of glucose; however, some tumor cells, such as glioblastoma (GBM), are classified as "glycolysis subtype" for the lack of ketone body oxidases, such as 3-hydroxybutyrate dehydrogenase (BDH1) or 3-succinyl-CoA transferase (OXCT1), because of genetic mutations or mitochondrial damage 31. In contrast, some cancer cells, such as breast or lung cancer cells, are classified as "ketone metabolic subtype" for their ability to use ketone bodies because of the high expression of ketone body oxidase 32. A study also revealed that "ketone metabolic subtype" tumor cells were significantly associated with catenin 1 (CTNNB1) gene mutation 33. Therefore, the use of ketone bodies instead of glucose as the main energy source can cut off the energy source of "glycolysis subtype" tumor cells with a minimal impact on the energy metabolism of immune cells, which provides a theoretical basis for the ketogenic diet (KD) therapy in such cases 34.

### Involved in signal transduction

Lipids are the precursors of signaling molecules (lipid mediators), which participate in intra- and extra-cellular signal transduction. Arachidonic acid (AA) derivatives, including prostaglandins (PGs), thromboxanes (TXs), and leukotrienes (LTs), mediate the evolution of chronic inflammation into the tumor 35, such as Barrett's esophagus to esophageal adenocarcinoma, gastric ulcer to gastric cancer, and pancreatitis to pancreatic cancer. PGs reduce oxidative stress and prevent lipid peroxidation in tumor cells 36. Notably, a study has established a paradigm for immune cell recruitment, described as lipid (PG and LT)-cytokine-chemokine cascade, as a driving force in the effector phase of immune responses 37. Therefore, targeting the PG synthesis pathway is a major approach for sensitizing immunotherapy.

Metabolites of PL, including inositol triphosphate (IP3), diacylglycerol (DAG), and lysophosphatidic acid (LPA), are associated with multiple oncogenic signaling pathways and tumor immune responses 15. IP3 promotes the release of intracellular Ca^2+^ and induce calcium overload, and DAG activates protein kinase C (PKC) leading to oxidative stress, thereby disrupting intracellular homeostasis. In addition, LPA binds to the G protein-coupled receptors (GPCRs) on the membranes of tumor and immune cells and activates RAS, RAC, and RHO signaling pathways 38.

Exosomes are one of the important forms of intercellular communication. Tumor cells can secrete numerous tumor-derived exosomes (TDEs) to transport signaling molecules to immune cells, thereby changing their metabolic state and inducing tumor immunosuppression. Lipids are the signaling molecules and also participate in the formation of the TDE membrane 39. TDEs have a bidirectional effect on tumor immunity. TDEs increase the accumulation of LDs in immune cells and promote their FAO, which contributes to the formation of an immunosuppressive microenvironment 40. Interestingly, TDEs carrying tumor biomolecules can mimic tumor antigens and shield the tumor cells from the attacks of the immune system or targeted drugs, which further promotes immune evasion and leads to treatment resistance 39. In contrast, TDEs can present tumor antigens to T cells directly or through antigen-presenting cells (APCs) and activate macrophages, neutrophils, and natural killer (NK) cells to release antitumor factors, thereby promoting antitumor immunity 41. Notably, immune cell-derived exosomes also induce an antitumor immune response. For example, exosomes secreted by APCs can stimulate the proliferation of T cells 42. Exosomes derived from M2 macrophages can transfer key enzymes of the PG pathway to tumor cells, such as cyclo-oxygenase 1 (COX1) and TXA synthase 1 43. Therefore, exosomes have great potential in tumor therapy including immunotherapy.

### Components of membrane structure

Lipids are important components of biological membranes and are involved in the transport of energy, materials, and information across membranes 44. In addition, special domains in the membrane, such as lipid rafts, play an important role in the communication between tumor and immune cells 45.

The type and saturation of FAs in the membrane affect its structural stability and function 46. On the one hand, saturated fatty acids (SFAs) are important for maintaining membrane fluidity and selective transmembrane transport. On the other hand, unsaturated fatty acids (UFAs), especially polyunsaturated fatty acids (PUFAs), are susceptible to reactive oxygen species (ROS), which results in lipid peroxidation and increases the sensitivity of cells to ferroptosis. In contrast, the peroxidation of monounsaturated fatty acids (MUFAs) plays a protective role 47. Therefore, the tumor cell membrane is characterized by an increased ratio of MUFA/SFA and MUFA/UFA 48. However, all PUFAs are not harmful to tumors. A study has reported that ω-3PUFA rather than ω-6PUFA has an antitumor effect, and the plasma levels of ω-3PUFA markedly decrease, whereas those of ω-6PUFA increase in patients with advanced tumors 49.

The key enzyme that regulates the type and saturation of FA in the membrane is stearoyl-CoA desaturase (SCD). Its Inhibition reduces the MUFA/SFA ratio and contributes to the induction of ferroptosis in tumor cells 47. However, fatty acid desaturase (FADS) can replace SCD in partially SCD1-dependent or SCD1-independent tumor cells such as liver and lung cancer cells. Inhibition of FADS1/2 reduced the ratio of MUFA/SFA and ω-3/ω-6PUFA. Notably, FADS2 also produced the uncommon MUFA sapienate, whose level was abnormally increased upon SCD1 inhibition 50. In addition, the PUFA/SFA ratio in the membrane is regulated by the remodeling of glycerol phosphatides (GPLs) remodeling through the Lands cycle 51. Tumor cells undergo significant GPL remodeling and phospholipase A2 and lysophosphatidylcholine acyltransferase (LPCAT) are the key enzymes of this process, which can be used as tumor markers and potential targets for therapy 52.

Lipid rafts are microdomains in cell membranes that are rich in cholesterol and sphingomyelin with long SFA chains; these chains contribute to the anchoring of LPs and the formation of relatively stable liquid regions. High levels of lipid rafts are present in the tumor cell membranes, where many tumorigenic proteins, such as CD44, epidermal growth factor receptor (EGFR), and insulin-like growth factor receptor are localized 53. Polyphenols can exert their antitumor effect by destroying lipid rafts 54. Interestingly, lipid rafts also localize immune-recognition receptors, such as T-cell receptor (TCR), major histocompatibility complex II (MHC II) 55, and transmembrane tumor necrosis factor-α (TNF-α), and provide a specialized environment for the activation of signaling complexes in response to antigen recognition 56.

### Involved in the modification of biomolecules

The great variability among lipids, in terms of their structure and properties, contributes to the remarkable diversity of lipid modifications. Among them, the most important is the acylation modification of proteins, in which FA is covalently linked to the peptide chain in the form of an acyl group 57. In addition to classical lysine acetylation, many novel acylation modifications of histone and non-histone proteins, such as malonylation (Kma, mediated by malonyl-CoA), β-hydroxybutyrylation (Kbhb, mediated by β-hydroxybutyric acid; BHB), isoprene (mediated by intermediates of cholesterol synthesis), and long-chain fatty acid (LCFA) acylation, have been identified 58. Histone acylation affects the epigenome through transcriptional regulation of gene expression, thereby linking epigenetics to lipid metabolism. Non-histone acylation mainly regulates protein stability, catalytic activity, membrane localization, and protein-protein interactions, thereby affecting protein function and linking lipid metabolism to the regulation of key proteins 57.

Metabolic reprogramming of tumor cells can lead to abnormal lipid modification. In turn, the changes in lipid modification affect tumor metabolism and the occurrence and development of tumors 59. For example, crotonylation (Kcr) -mediated DNA damage repair and spindle localization help maintain genome integrity and prevent tumor progression. In addition, p53 can be modified by lipid modifications such as Kbhb, succinylation, propionylation, butyrylation, and Kcr, leading to decreased expression of p53 and its downstream genes and affecting p53-mediated tumor suppression. Notably, palmitoylation of programmed cell death ligand 1 (PD-L1) helps to maintain its stability, thereby inhibiting the cytotoxicity of CD8+T cells. In addition, palmitoylation also regulates tumor innate immunity and inflammatory responses 60.

## Landscape of abnormal lipid metabolism in tumors

**Figure 1:**
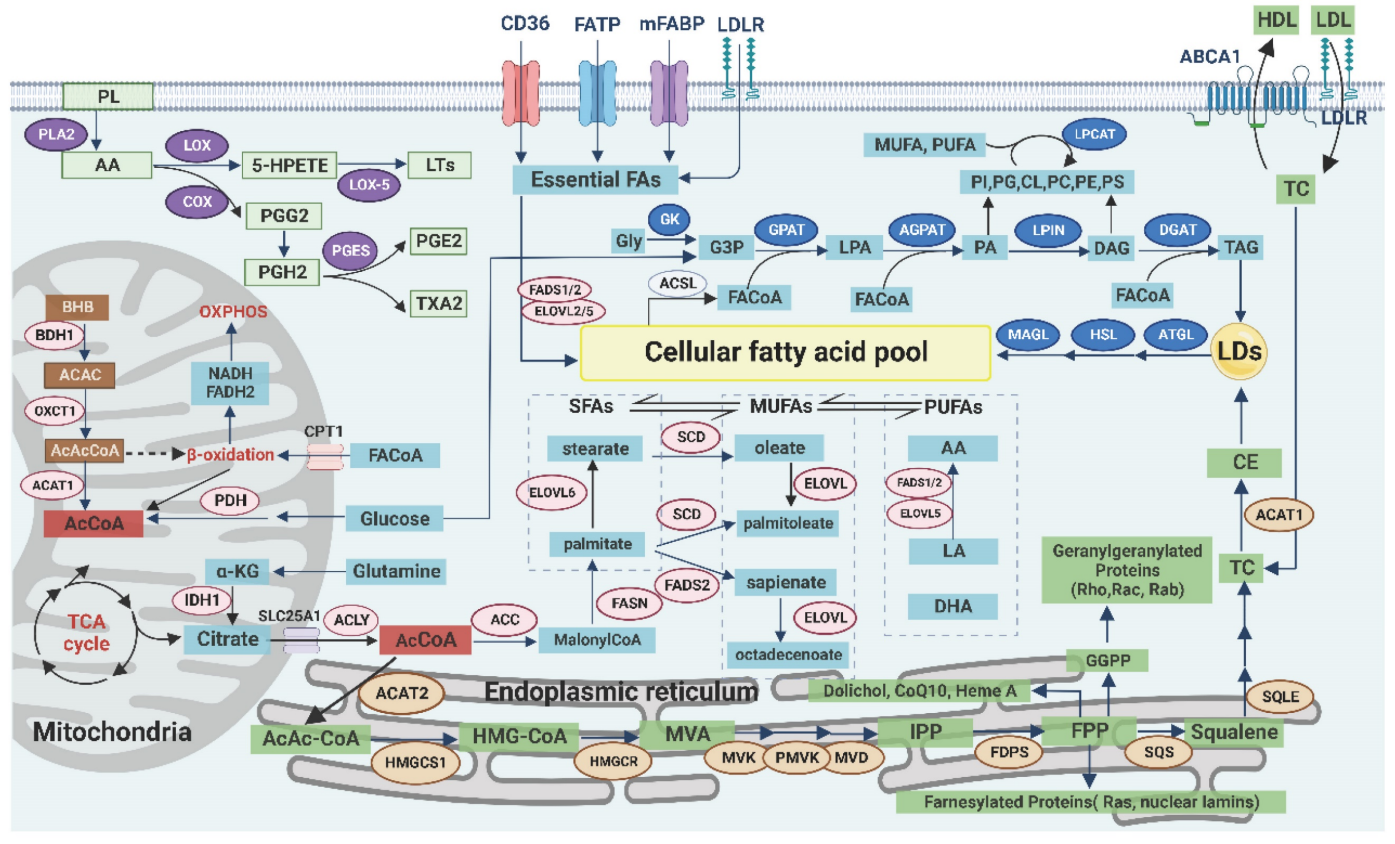
** Landscape of lipid metabolism.** In the membrane, AA released from PL participates in the PG synthesis pathway, which affects inflammation and redox balance. The transmembrane transport of exogenous lipids, especially essential FAs, can be achieved through various receptors to form MUFAs. In the mitochondria, AcCoA can be generated from glucose, glutamine, acetate, or ketone bodies and subsequently enter the cytoplasm to continue the FA synthesis. In the endoplasmic reticulum, AcCoA is involved in the synthesis of TC, and its intermediates are associated with lipid modification and redox balance. In the cytoplasm, the products of the de novo synthesis of FA are SFAs, which are further elongated and desaturated to form MUFAs. The excess lipids are stored in LDs as TAG and CE, whereas FAs are used as substrates for lipid remodeling via the Lands cycle and then released when needed.

Lipid metabolism, especially anabolism, is highly active in tumor cells to promote their survival and development in the TME, which is characterized by the high expression of receptors and key enzymes involved in lipid uptake, synthesis, storage, and decomposition. However, the expression of key enzymes is different because of the different types, stages, and microenvironments of tumors (Table 1, Figure 1) 61.

### Exogenous uptake of lipids

The supply of exogenous lipids is easily affected by dietary intake. The occurrence and development of tumors are closely related to the amount and type of lipids consumed. High-fat diets (HFD, mainly containing SFA and cholesterol) can increase the risk of cancer 62, and a clinical study has found that lipid-lowering drugs can improve the prognosis of patients with cancer 63. However, not all SFA are harmful. For example, palmitic acid can induce apoptosis of tumor cells under certain conditions 64. In contrast, people who increase the intake of PUFA (specifically ω-3PUFA) have a reduced risk for cancer 65. ω-3PUFA promotes lipid peroxidation and induces ferroptosis in tumor cells in the acidic TME 66. However, not all PUFAs have antitumor effects. For example, high levels of ω-6PUFA intake combined with low levels of ω-3PUFA intake increase the risk of cancer 67. This partially happens because the short-chain adducts derived from ω-3PUFA oxidation are less likely to cause mispairing of DNA, whereas the long-chain ones from ω-6PUFA preferentially bind to p53, thereby affecting p53-mediated tumor suppression and inducing carcinogenesis. ω-3PUFA competes with ω-6PUFA to bind COX and lipoxygenase (LOX) and reduces the generation of PGs; ω-3PUFA can also regulate autophagy and inhibit the NF-kB pathway 68. Therefore, changes in dietary lipid intake can be a selective antitumor therapy and provide a new strategy for sensitizing immunotherapy.

The transmembrane transport of exogenous lipids can be achieved through a variety of pathways. Low-density lipoprotein (LDL) enters the cell through endocytosis mediated by LDL receptor (LDLR), and it is subsequently digested by lysosomal acid lipase (LAL) to release free fatty acids (FFA) and cholesterol. LDLR is highly expressed in tumors and enhances the EGFR, PI3K/Akt, and sterol regulatory element-binding protein (SREBP) signaling pathways. Tumor metastasis is also associated with persistently high expression of LDLR 69. LCFA can be transported by fatty acid translocase (CD36, belonging to scavenger receptor) or fatty acid transporter (FATP, belonging to solute carrier family SLC27A) 70. In addition, CD36 mediates the uptake of oxidized low-density lipoprotein (ox-LDL) and cholesterol. The high expression or structural abnormalities of CD36 were closely related to tumor growth, invasion, and metastasis. CD36 promotes FAO, angiogenesis, and tumor chemoradiotherapy resistance by regulating the Src/PI3K/Akt signaling pathway. CD36 can also activate Wnt/TGF-β signaling pathway through epithelial-to-mesenchymal transition (EMT) to promote metastasis 71. Plasma membrane fatty acid binding proteins (FABPpm) contribute to FA uptake, whereas intracellular FABPs mediate the transport of FA within the cell. The potential protumor role of FABP4 has been demonstrated, and inhibition of FABP affects tumor growth and invasion. Therefore, FABP has been used as a marker for colorectal cancer (CRC) and renal cell carcinoma (RCC) 61, 72. The ability of tumor cells to absorb lipids is affected by oncogene types, and not all lipids are taken up equally. For example, tumor cells selectively take up specific lysophospholipids (lysoPLs, PL containing MUFA, and PUFA acyl chains) from the TME 73. Similarly, tumor-associated immune cells can selectively take up lipids according to the state of differentiation and activation but can also passively ingest some harmful lipids in the TME, such as ox-LDL 74.

### Synthesis of lipids

#### De novo synthesis and desaturation of fatty acids

De novo synthesis of FA begins with acetyl coenzyme A (AcCoA), which is mainly produced by glucose, glutamine 75, and acetate 76 in the cytoplasm. The citric acid in mitochondria is transported into cells through SLC25A1 (carrier protein), and then AcCoA is generated by ATP-citrate lyase (ACLY) 20. SLC25A1 plays a key role in the maintenance of the mitochondrial citric acid pool and redox balance, and its inhibition leads to the accumulation of ROS 77. ACLY promotes tumor invasion and metastasis by regulating the Wnt/β-catenin signaling pathway 78. Additionally, ACLY promotes the early activation of CD8+T cells and their lipid metabolic reprogramming 79. In addition to being involved in the synthesis of FA and cholesterol, AcCoA participates in acylation modifications of proteins, and its expression level is dynamically associated with histone acetylation 80. The expression of AcCoA is also related to programmed cell death and autophagy. Therefore, targeting AcCoA metabolism has potential in tumor treatment and may also have implications in immunotherapy 81.

Acetyl-CoA carboxylase (ACC/ACAC) catalyzes the conversion of AcCoA to malonyl-CoA, which can inhibit carnitine palmitoyltransferase 1 (CPT1)-mediated FAO in mitochondria, thereby regulating the balance between synthesis and decomposition of FA. In addition, activated AMP-activated protein kinase (AMPK) inhibits the activity of ACC1/2 82. Then, fatty acid synthase (FASN) catalyzes the conversion of malonyl-CoA to palmitic acid. In addition to being involved in synthesis of FA, FASN can impact other metabolic pathways such as glycolysis, amino acid metabolism, and ferroptosis 83. Therefore, FASN and other key enzymes can be used as potential diagnostic markers for molecular subsets of tumors with high levels of lipid metabolism 84.

Palmitic acid is subsequently elongated by the activity of elongation of the very long chain fatty acid (ELOVL) family of enzymes and converted to MUFA by SCD1. SCD1 can relieve the inhibitory effect of SFAs (including palmitic acid) on the activity of upstream ACC by reducing the SFA levels and subsequently increasing the production of malonyl-CoA, which affects FAO 26. In addition to affecting the membrane composition, SCD1 can damage the mitochondrial electron transport chain (ETC) and reduce the binding of MUFA to cardiolipin, which increases the production of intracellular ROS and induces mitochondria-dependent apoptosis. Inhibition of SCD1 also leads to endoplasmic reticulum stress, which, in turn, induces antitumor immunity 85.

Furthermore, SCD1 affects the sensitivity of the cells to ferroptosis. On the one hand, inhibition of SCD decreases the MUFA/UFA in the membrane, and MUFA has an antiperoxidative effect, which, in turn, promotes ferroptosis 47. On the other hand, inhibition of SCD1/FADS2 directly downregulates key enzymes and proteins, such as glutathione peroxidase 4 (GPX4) and reduced glutathione (GSH), respectively, in the ferroptosis-related antioxidant defense system, and disrupts cellular/mitochondrial redox balance, which leads to iron-mediated lipid peroxidation and results in mitochondrial dysfunction 86.

#### De novo synthesis of cholesterol

A high level of cholesterol is essential for the proliferation and metastasis of tumor cells to fulfill the requirement for membrane structure. cholesterol also stabilizes PD-L1 on the tumor cell surface, thus inhibiting tumor immunity 87. In addition, high levels of cholesterol in the TME also affect the phenotype and activity of tumor-associated immune cells 88.

Cholesterol is primarily synthesized through the mevalonate (MVA) pathway. The key enzyme is HMG-CoA reductase (HMGCR), which is highly expressed in many cancers, and targeted inhibition of this enzyme has emerged as a potential therapeutic approach 89. In addition to the end product cholesterol, the intermediates of the MVA pathway are equally important for the survival and development of tumor cells and antitumor immune response including ferroptosis 90. For example, phosphate polyterpene alcohols are essential for the synthesis of lipid-linked oligosaccharides and are used for the N-glycosylation of proteins 91. Farnicyl pyrophosphate and geranyl pyrophosphate, as isoprene derivatives, participate in the isoprenylation modification of protein fa such as Ras and Rho. They can also upregulate the expression of PD-1 on the surface of immune cells such as regulatory T cells (Treg cells) 92. Coenzyme Q10 (CoQ10/ubiquinone) is a coenzyme of cytochrome C oxidase in the mitochondrial respiratory chain complex IV. Under the metabolic stress of the TME, impairment of the respiratory chain by inhibiting the expression of CoQ10 can cause tumor cell death. In addition, CoQ10 inhibits iron action suppressor protein 1 (FSP1), consequently suppressing ferroptosis. This process is positively correlated with the activity of SCD1 93. Squalene acts as a free radical trapping antioxidant to protect membrane lipids from peroxidation 90. Isopentenylpyrophosphate is required for the maturation of selenocysteinyl-tRNA and GPX4 synthesis, which, in turn, inhibits ferroptosis 94. Taken together, these findings suggest that de novo cholesterol synthesis may play a protective role against ferroptosis in tumor cells.

### Storage of lipids

Excess lipids are stored in LDs in the form of TAG and cholesterol ester (CE). The number of LDs and the amount of stored TAG and CE inside them abnormally increases in tumor cells and tumor-associated immune cells (especially macrophages) 95. The low pH of the TME enhances the formation of LDs in tumor cells 96. Therefore, LD accumulation is considered a prominent feature of tumors. Phosphoenolpyruvate carboxykinase (PCK1), the key enzyme in the gluconeogenesis pathway, promotes the continuous formation of LDs through the activation of the SREBP signaling pathway in human hepatocellular carcinoma (HCC) cells 12, 97.

The core of an LD consists of neutral fats (mainly including TAG and CE) and is surrounded by a single layer of PLs and several proteins. Various enzymes involved in the synthesis of TAG and lipolysis are localized on the surface of LDs 98. In addition, LDs have a selective form of autophagy (lipophagy). Therefore, LDs not only simply store lipids but also actively participate in the lipid metabolism pathway. Furthermore, the formation or alteration of LDs is tightly regulated by the cell to meet the requirements of lipid metabolism.

LD is a dynamically changing and multifunctional organelle 96. As a reservoir of lipids, LD not only protects its contents from enzymatic decomposition but also protects the cell from the toxic effects of excess FFA, cholesterol, and ROS 99. Especially for immune cells in the acidic and lipid-rich TME, LD mediates the flow of cholesterol to membrane, thereby regulating the intracellular distribution of cholesterol, and helps cholesterol participate in the formation of immune synapse 100. In addition, LDs move along the cytoskeleton and interact with other organelles, thereby playing an important role in transmembrane transport, protein degradation, and signal transduction 99. LDs also act as a hub of host innate immunity and affect the tumor immune response 101.

### Decomposition of lipids

#### Decomposition of fatty acids

The main function of FAO, known as β-oxidation, is to provide energy. In addition, it serves as a source of NADPH especially when glucose is deficient or the pentose phosphate pathway (PPP, mainly producing NADPH) is compromised 102. FAO is highly active in many tumor cells, such as lung cancer cells with K-Ras mutation 103 and tumor-associated immune cells, indicating their adaptation to the metabolic stress of TME. CPT1 is critical for the transport of LCFAs into the mitochondria for β-oxidation. However, FAs need to be activated by the attachment of CoA through the activity of long-chain acyl-CoA synthetase 3 (ACSL3), leading to the formation of FA-CoA 104. In addition, ACSL participates in TAG synthesis, autophagy, the Lands cycle, and ferroptosis and promotes the proliferation and progression of tumors. Therefore, ACSL can be a potential marker and therapeutic target for tumors 105.

#### Decomposition of cholesterol

The basic structure of cholesterol is cyclopentane polyhydrophenanthrene, which cannot be degraded completely. However, its oxidative metabolites, such as 27-hydroxycholesterol (27-HC; an oxysterol), affect tumor development. In the TME, 27-HC is mainly derived from macrophages and endothelial cells. 27-HC inhibits the uptake and synthesis of FA and cholesterol by inhibiting the SREBP pathway and activating the liver X receptor (LXR) pathway 106. In contrast, 27-HC can competitively inhibit the effect of estrogen because of its selective estrogen receptor (ER) regulatory function and promotes the proliferation of high-ER-expressing tumor cells, such as breast and ovarian cancer cells 107. It can also induce an increase in GPX4 expression, thereby protecting tumor cells from ferroptosis. This phenomenon partially explains why metastatic breast tumor cells are not damaged by ferroptosis induced by lipid accumulation while taking up lipids in large quantities 108.

#### Decomposition of ketone bodies

The catabolism of ketone bodies in tumor cells of the "ketone metabolic subtype" serves as an important energy source 32. The ketone bodies, especially BHB, can serve as a negative feedback signal to promote lipid metabolism and regulate the tricarboxylic acid (TCA) cycle. For example, BHB decreases the LDL level and increases the high-density lipoprotein level. As a key endogenous GPCR signaling modulator, BHB can also inhibit the process of fat mobilization by activating GPCR109A and reducing the supply of extracellular lipids 109. In addition, BHB can regulate the respiratory chain and upregulate genes involved in protecting cells from oxidative stress as an endogenous inhibitor of histone deacetylase. Notably, BHB also promotes tumor immune response by downregulating the PD-L1 levels and promoting the proliferation of T cells 109.

## Abnormal regulation of lipid metabolism

**Figure 2:**
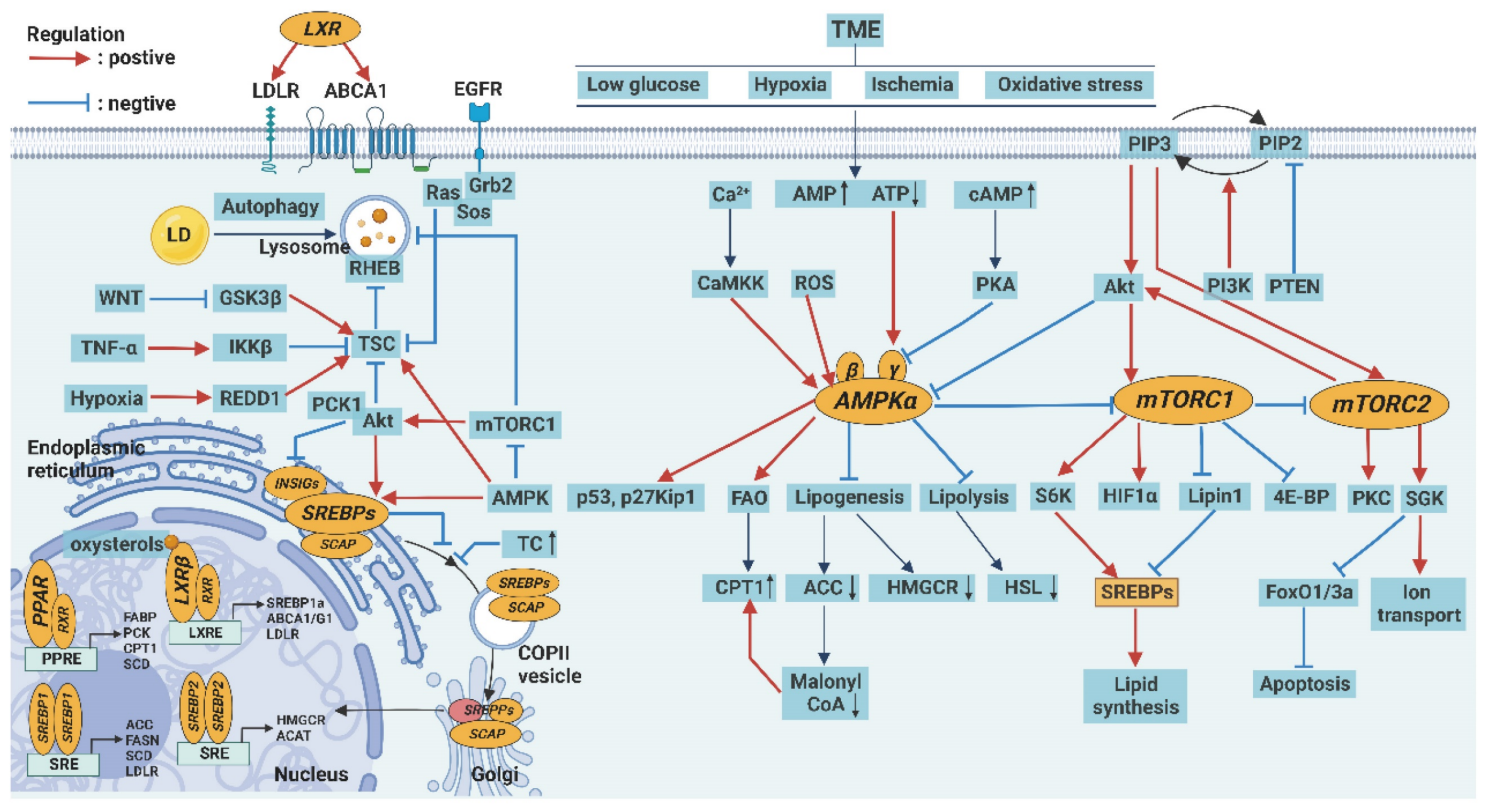
** Regulation of lipid metabolism.** The expression of various key enzymes of lipid metabolism can be regulated by signaling pathways, including AMPK, mTOR, PPAR, SREBP, and LXR signaling pathways. In the endoplasmic reticulum, SREBPs, as inactive precursors, reside as transmembrane proteins where they are associated with a chaperone, the SREBP cleavage activating protein (SCAP). When intracellular cholesterol levels are reduced, SREBPs are transported to the Golgi, where a two-step proteolytic process releases the N-terminal half of the protein, and then mature SREBPs translocate to the nucleus. In the nucleus, transcriptional regulators that control the synthesis and transport of FA and TC are SREBPs, LXRs, and PPARs, which bind as homodimers or heterodimers to SREs, LXRE, and PPREs, respectively, in the promoter regions of their target genes. In the TME, energy stress, increased Ca2^+^ level, and oxidative stress increase AMP/ATP or ADP/ATP ratio to activate AMPK but inhibit mTOR, which promotes autophagy.

The expression of various key enzymes in lipid metabolism can be regulated by several signaling pathways, including AMPK and mammalian target of rapamycin (mTOR), peroxisome proliferator-activated receptors (PPAR), SREBP, and LXR signaling pathways. These pathways are further regulated by upstream signaling pathways such as p53, BRAF, PTEN, Ras, and Myc 110. However, the lipid metabolic pathway is not a passive recipient of signals. Some metabolic intermediates can feedback information about the metabolic state of cells to the genes and proteins responsible for regulation through histone acylation modification 111. The close relationship between the lipid metabolism and signal regulation networks plays an important role in the survival and growth of tumor cells and tumor-associated immune cells (Figure 2).

### AMPK and mTOR signaling pathways

AMPK and mTOR are mutually antagonistic key molecules in the regulation of energy metabolism. The factors, such as energy stress, increased Ca^2+^ level, and oxidative stress increase AMP/ATP or ADP/ATP ratio and activate AMPK in cells, which promote catabolism and inhibit anabolism and confer resistance to metabolic stress 112.

Specifically, AMPK enhances FA uptake through the upregulation of CD36 and other lipid transporters and inhibits SREBP signaling. In addition, AMPK activates autophagy directly or indirectly by inhibiting mTORC1 113. AMPK activation also inhibits cell proliferation by promoting the p53 pathway and inhibiting the cyclin-dependent kinase (CDK) pathway 112. Notably, loss of AMPK in Treg cells promotes tumor immunosuppression by increasing the expression of PD-1 114. In contrast, the activation of AMPK in NK cells mediates antitumor immunity and enhances the effect of anti-PD-L1 therapy 115. Therefore, AMPK is considered an important tumor suppressor.

However, AMPK can also promote tumor growth and metastasis in some cases. For example, AMPK can prevent lipid peroxidation and inhibit antitumor immune responses by activating the production of antioxidants and inhibiting inflammatory responses through the NF-κB pathway 116.

When nutrients are sufficient, mTOR is activated instead of AMPK to promote anabolism and inhibit catabolism. mTORC1 and mTORC2 are two different complexes in the cell. mTORC1 promotes anabolism by activating ribosomal protein S6K (S6K) and inhibiting EIF4E-binding protein (4EBP) and is sensitive to rapamycin, whereas the mTORC2 promotes cell survival and proliferation by activating protein kinases such as AKT, SGK, and PKC and is not sensitive to rapamycin 117. In addition to regulating metabolism, S6K and AKT can activate hypoxia inducible factor 1 α (HIF-1α) and SREBP1, thus promoting angiogenesis and FA synthesis 118. mTOR also inhibits apoptosis, autophagy, and ferroptosis 119.

However, over-activation of mTOR can accelerate cell senescence and even induce cell death. Contrary to AMPK, mTORC1 induces mitochondrial oxidative stress by inhibiting the transcription factor PGC-1α, which promotes lipid peroxidation and calcium overload 120. The continuous activation of mTOR can also disrupt the balance of pro- and anti-inflammatory immune responses, leading to the generation of chronic inflammation 121.

### PPAR signaling pathway

PPARs are ligand-induced nuclear receptors and are involved in several peroxide proliferation reactions, including metabolic and apoptotic pathways related to lipid metabolism. PPARs are generally highly expressed on tumor cells, and their expression is dependent on several factors such as PPAR subtype and type and stage of the tumor 122. When activated by ligands such as AA derivatives, PUFA, and non-steroidal anti-inflammatory drugs (NSAIDs), PPARs receptors enter the nucleus to regulate the transcription of genes encoding enzymes of lipid metabolism, such as FABP, CPT1, and SCD1, and peroxisome genes related to lipid peroxidation 123. A zinc-finger protein interacts with PPARs to regulate their activity. Zinc deficiency upregulates the expression of PPARc, FABP, and SREBP1, which results in lipid accumulation 124. In addition, PPARγ can induce apoptosis of tumor cells through the exogenous Fas/FasL death receptor pathway or endogenous mitochondrial apoptosis pathway 125. Notably, PPARγ regulates the innate immune system by promoting the apoptosis of N1 neutrophils and activating M2 macrophages. PPARα also plays an important role in inhibiting inflammatory pathways such as NF-κB and JAK/STAT pathways 126. Therefore, the PPAR signaling pathway can be a potential target for tumor immunotherapy.

### SREBP and LXR signaling pathways

SREBPs are members of the nuclear transcription factor family. SREBP1 regulates FA metabolism 127, whereas SREBP2 regulates cholesterol metabolism 128. When the level of intracellular lipid decreases, the precursor proteins of SREBPs residing on the endoplasmic reticulum membrane are transported to the Golgi apparatus, where they are cleaved, released into the nucleus, and promote the expression of genes encoding key enzymes of lipid metabolism. SREBP1 promotes ACC, FANS, and SCD, whereas SREBP2 promotes HMGCR and ACAT 90.

The SREBP signaling pathway is abnormally activated in tumor and immune cells. Abnormally activated intracellular PI3K/Akt/mTOR signaling pathway promotes the expression of SREBP1 129. TP53 gene mutations can also promote the activation of the SREBP signaling pathway 130. In addition, SREBP signaling interacts with glucose metabolism pathways, thereby linking glucose and lipid metabolism. Pyruvate kinase M2 (PKM2), a key enzyme in glycolysis, modifies SREBP1a to enhance its stability 131. In turn, SREBP1a also promoted the expression of isocitrate dehydrogenase 1 (IDH1), a key enzyme of the TCA cycle, by binding to its promoter region 132. Notably, SREBP2 is involved in the inflammatory response associated with Nod-like receptor protein 3 (NLRP3), which affects the tumor immune response 133.

Similarly, LXRs synergize with SREBPs as key regulators of cholesterol homeostasis in cells. LXRs are activated by intracellular cholesterol or 27-HC and subsequently promote transcription of target genes encoding ATP-binding cassette transporter A1/G1 (ABCA1/G1), SREBP1a, and LDLR 134. When the intracellular cholesterol level is too high, LXRs inhibit the SREBP2 signaling pathway. They promote the efflux of cholesterol by increasing the expression of ABCA1/G1 and apolipoprotein A1/E (ApoA1/E) and inhibit the uptake of cholesterol by enhancing the degradation of LDLR 135. Activation of LXRs can also induce tumor cell apoptosis by blocking key growth pathways such as the EGFR pathway and downregulating survival signals such as Akt 136. Notably, the activation of LXR receptors induced the differentiation of macrophages into an M2-like phenotype 137.

## Lipid metabolic reprogramming in tumor-associated immune cells

Immune cells can recognize and destroy tumor cells during immune surveillance. However, in the process of tumor immune editing, the products of tumor lipid metabolism not only modify the TME but also affect the recruitment and function of tumor-associated immune cells. Therefore, immune cells can act as guardians (antitumor immunity) as well as bystanders or even supporters (protumor immunity) 138.

Immune cells can be divided into two types based on their effect on tumors. Antitumor immune cells include effector CD8+T (Teff) cells, antitumor CD4+ (CD4+conv) cells, memory CD8+T (Tmem) cells, B cells, natural killer T (NKT) cells, NK cells, M1 macrophages, dendritic cells (DCs), and N1 neutrophils. Protumor immune cells include Treg cells, regulatory B (Breg) cells, M2 macrophages, myeloid-derived suppressor cells (MDSCs), and N2 neutrophils. Lipid metabolic reprogramming plays a crucial role in regulating the phenotype and function of immune cells. Although certain types of antitumor/protumor cells rely on both types of metabolism, in general, antitumor immune cells tend to rely on glucose metabolism to exert anti-tumor function, whereas protumor immune cells tend to rely on lipid metabolism to exert tumor immunosuppressive function (Table 2) 25, 139-141.

### Abnormal metabolism of antitumor immune cells

#### Adaptive immunity: Teff, CD4+conv, Tmem, and B cells

Teff cells directly kill tumor cells by inducing apoptosis and secreting cytokines, such as interferon-γ (IFN-γ) and TNF-α, and CD4+conv cells mainly exert an indirect antitumor effect by assisting Teff cells. Teff cells require an adequate supply of glucose for their antitumor functions. Although enhanced glycolysis is critical for T-cell activation, the upregulation of the TCA cycle and OXPHOS is equally important, and specifically, FAO also maintains the antitumor function of Teff cells 142. In addition, lipid metabolism is enhanced in activated T cells leading to an increase in FA synthesis and cholesterol uptake, which contributes to the maintenance of membrane structure and immune receptor signaling.

Similarly, the metabolic bypass of glycolysis, that is PPP, is also upregulated with the activation of T cells to provide NADPH for lipid synthesis. However, the dependence of CD4+T cells on FAO promotes their differentiation into Treg cells 138. The activation of T cells depends on the mitochondrial activity (similar to tumor cells), mitochondria in tumor-associated T cells are defective, which predisposes to intracellular oxidative stress and impairs their antitumor effect 143.

Notably, CD8+T cells can generate a memory cell population (Tmem cells), which can persist after the tumor response fades; therefore, these cells are the key to long-term tumor control. Unlike Teff cells, Tmem cells, as quiescent cells, preferentially rely on FAO instead of glycolysis. These cells have significant mitochondrial reserves of FAs and further upregulate OXPHOS after activation 139. Under the metabolic stress of the TME, Tmem cells tend to increase their uptake of FAs and enhance acetate metabolism to promote lipid synthesis, thereby reversing the apoptosis induced by lipid deprivation and retaining their antitumor function 144.

B cells differentiate into plasma cells and produce tumor-specific antibodies to recognize and mediate tumor lysis through antibody-dependent cytotoxicity (ADCC) or complement-dependent cytotoxicity. B cells also promote the activation of T cells. In addition, B cells can directly kill tumor cells by secreting granzyme B and tumor necrosis factor-related apoptosis-inducing ligand 145. Toll-like receptor (TLR) and B-cell receptor (BCR) signaling can lead to the activation of B cells and increase oxygen consumption and glucose transport 146. However, unlike T cells, B cells mainly utilize glucose through PPP to produce NADPH and ribose 5-phosphate, which are required for the synthesis of antibodies. In addition, activation of TLR and BCR signaling induces differentiation of Breg cells and promotes their glycolytic metabolism 147.

#### Innate immunity: NKT cells, NK cells, M1 macrophages, DCs, and N1 neutrophils

NKT cells have the characteristics of both adaptive and innate immune cells and mainly recognize lipid antigens to play an immunomodulatory role. Therefore, lipid metabolism can promote the activation of NKT cells and regulate their cytotoxic effect. High levels of lactic acid in the TME reduced the expression of PPARγ in NKT cells, thereby decreasing lipid synthesis and production of IFN-γ. In addition, high levels of cholesterol in the TME can promote the secretion of IFN -γ by NKT cells and enhance the TCR signaling at immunological synapses of NKT cells 139, 148.

NK cells can directly kill tumor cells by releasing cytotoxic granules and inflammatory cytokines or through ADCC. Glycolysis and OXPHOS are upregulated during the activation of NK cells, which is mediated by Myc, SREBP, and mTORC1 signaling pathways. Myc is regulated by mTORC1 and promotes glycolysis and OXPHOS in NK cells by promoting the expression of glucose transporter protein (GLUT) and key enzymes of glycolysis 140. However, an increase in endogenous SREBP inhibitors such as 27-HC in the TME inhibits glycolysis and OXPHOS in NK cells by reducing the expression of SLC25A1 and ACLY; moreover, the secretion of IFN-γ and granzyme-B is also inhibited. In addition, glucose-1, 6-bisphosphatase (FBP), the key enzyme of gluconeogenesis, significantly inhibits glycolysis in NK cells and leads to their dysfunction 149.

M1 macrophages exert antitumor immune responses by producing pro-inflammatory cytokines and inducible nitric oxide synthase and participating in ADCC. When TLR is activated, M1 macrophages show increased glucose uptake and lactic acid production. However, the lack of glucose and the accumulation of lactic acids in the TME promotes the transformation of macrophages from antitumor M1 to protumor M2 phenotype. In addition, M1 macrophages depend on NADPH produced through PPP. In the TME, PUFAs promote the aggregation of M1 macrophages accompanied by an increase in ROS, while SFAs inhibit the antitumor function of M1 macrophages. Notably, exogenously modified lipids, such as phosphotyrosine cholesterol, can polarize or even re-induce the antitumor M1 phenotype 150, 151.

DCs, an important APC in antitumor response, switch from OXPHOS to glycolysis after activation. The transition process is mainly related to LPS/HIF-1α and TLR/PI3K/AKT pathways. In the lipid-rich TME, DCs tend to accumulate PL and TAG (especially PUFA) rather than cholesterol and CE, which enhances the secretion of pro-inflammatory cytokines and chemokines, thereby playing a stronger antigen cross-presentation function and effectively activating NK, NKT, and Teff cells. In addition, 27-HC was found to induce the maturation of DCs. However, high levels of oxidized lipids and high accumulation of LDs limit the formation of the MHC I complex and impair the ability of DCs to present antigens and activate T cells. In addition, liver kinase B1 is an important metabolic regulator of DCs and the loss of its expression can activate the mTOR signaling pathway, resulting in metabolic disorders in DCs 151, 152.

N1 neutrophils can exert antitumor effects by producing ROS, reactive nitrogen species, and IFN-α/β and phagocytosis. They can activate NK cells and inhibit IL-17-producing T cells 153. N1 neutrophils mainly rely on glycolysis, and they also upregulate PPP to meet the NADPH requirement for respiratory burst. In addition, N1 neutrophils have active glycogen metabolism to fulfill their antitumor function 154.

### Abnormal metabolism of protumor immune cells

#### Adaptive immunity: Treg and Breg cells

Treg cells are the key cell population involved in adaptive tumor immunosuppression. Treg cells secrete inhibitory factors such as IL-4, IL-10, and transforming growth factor-β (TGF-β) and express inhibitory molecules such as CTLA-4, PD-1, and LAG-3 to inhibit the activation and function of other immune cells. Treg cells preferentially utilize the TCA cycle and OXPHOS for their metabolism. However, Treg cells are more dependent on FAO under the metabolic stress of the TME. In contrast to Teff cells, Treg cells show lower levels of GLUT1 and higher levels of monocarboxylate transporter 1 (MCT1), CD36, and FANS, which increase lactic acid and lipid metabolism to replace glucose metabolism. Similarly, the expression of genes and proteins related to glucose metabolism is downregulated in Treg cells, which are activated by TLR8 141. In addition, the activation of the AMPK and SREBP pathways enhances the synthesis of FA and cholesterol, which, in turn, increases the expression of PD-1 in Treg cells 114. However, the activity of ACLY decreases significantly during the differentiation of Treg cells stimulated by TGF-β, which leads to the transformation from lipid anabolism to catabolism 155. Of note, some highly active Treg cells still rely on glycolysis. Therefore, Treg cells have good metabolic adaptability, which is conducive to their survival under the metabolic stress of the TME 156.

Breg cells also have an immunosuppressive function and inhibit the activation of T and NK cells. They also promote the protumor effect of Treg cells, MDSCs, and M2 macrophages by secreting IL-10, TGF-β, and ROS and upregulating PD-L1 and CTLA-4 157. High levels of lactic acid in the TME can induce the differentiation of antitumor B cells into protumor Breg cells through the GPR81/PPARs/NADPH oxidase signaling pathway 158. Like other B cell populations, glycolysis is enhanced in activated Breg cells, and the hypoxic TME and HIF-1α can maintain the function of Breg cells by promoting glycolysis. In addition, changes in cholesterol metabolism in B cells regulate IL-10 production 159.

#### Innate immunity: M2 macrophages, MDSCs, and N2 neutrophils

The phenotype of tumor-associated macrophages (TAMs) is mainly the immunosuppressive M2 phenotype. M2 macrophages secrete various protumor factors such as arginase 1 (Arg1). M2 macrophages, like Treg cells, are mainly dependent on FAO. M2 macrophages tend to take up more lipids from the TME and enhance the synthesis of FAs to maintain their protumor function. For example, oleic acid can induce M2 polarization through the mTOR signaling pathway 138. Cholesterol is an inflammatory mediator in M2 macrophages and ABCG1 (a transporter protein involved in cholesterol homeostasis and efflux) is highly expressed in M2 macrophages, thereby promoting cholesterol efflux and IL-4 secretion. In addition, 27-HC induces polarization of macrophages to the M2 phenotype, which may be related to the activation of LXRs. Besides, hypoxia increases the infiltration of M2 macrophages and inhibits M1 macrophages by producing angiogenic factors, mitogenic factors, and adenosine. Similarly, lactic acid in the TME enhanced the activity of HIF-2α by inhibiting the expression of the M2 macrophage-specific vacuolar ATP subunit (ATP6V0d2). Lactic acid also upregulates HIF-1α stable lncRNA in M2 macrophages 150, 160.

MDSCs are also important immunosuppressive cells that establish a complex tumor immunosuppressive network with other immune cells. Although glycolysis and OXPHOS are upregulated in MDSCs, these cells rely on FAO to maintain their protumor function 161. Serine/threonine kinase (PIM1) regulates FAO through PPARγ. The lipid-rich TME (having 27-HC) can promote the survival and infiltration of MDSCs, and these cells also upregulate CD36 and LDLR to further increase lipid uptake. Similarly, tumor-derived granulocyte-macrophage colony-stimulating factor (GM-CSF) promotes lipid accumulation in MDSCs by inducing the expression of FATP2 through STAT3 signaling. However, excessive lipid accumulation inhibits their immunosuppressive function. UFAs promote the formation of LDs, and LDs inhibit the activity of MDSCs 162, 163.

Tumor-associated neutrophils (TANs) have protumor N2 phenotype. These neutrophils secrete Arg1, myeloperoxidase, neutrophil elastase, matrix metalloproteases, and neutrophil extracellular traps to promote tumor development and inhibit the activation and proliferation of T and NK cells 153. Tumors can enhance the FAO and maintain intracellular redox balance in N2 neutrophils through the stem cell factor/c-Kit signaling pathway, which allows them to exert their immunosuppressive effects for a longer period. Cholesterol and oxysterols such as 27-HC promote the aggregation of N2 neutrophils and the formation of an immunosuppressive microenvironment in the TME 139. In addition, N2 neutrophils show a high expression of genes associated with lipid uptake and LD formation, and a low expression of genes related to the degradation of LDs and β-oxidation, which results in lipid accumulation in them. N2 neutrophils also demonstrate high expression of GLUT1. Notably, the accumulation of nutrients in N2 neutrophils may be related to the prolongation of their life span, thereby allowing them to exert immunosuppressive effects in the TME for a longer duration 164.

## Effect of lipid metabolic reprogramming on the TME

**Figure 3:**
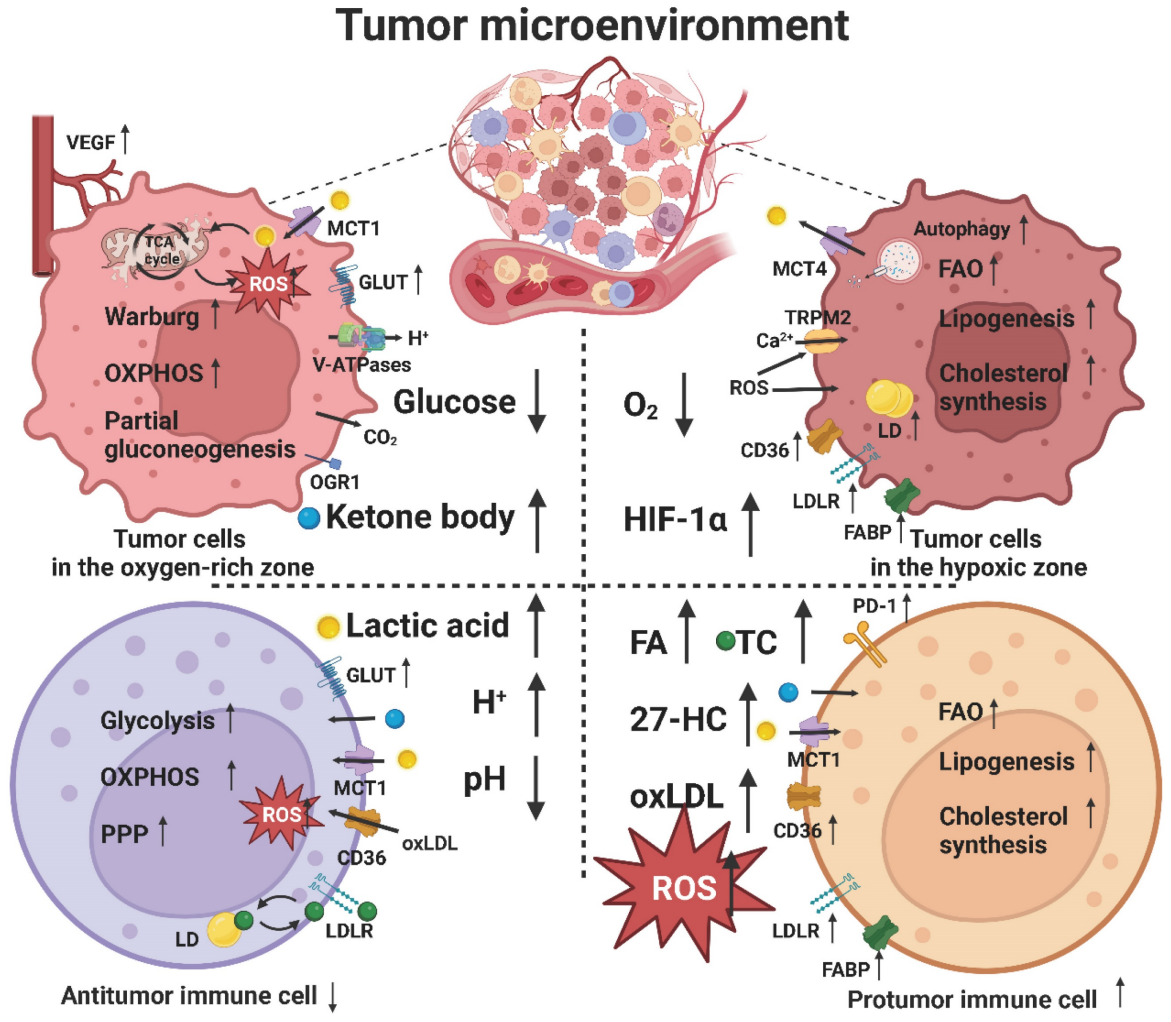
** Lipid metabolic reprogramming in the TME.** Metabolic stresses, such as glucose and oxygen deprivation, lactic acid accumulation, and lipid peroxidation, induce lipid metabolic reprogramming of tumor and immune cells in the TME to circumvent these limitations.

The TME plays an important role in tumor immunity and immunotherapy. Tumor cells can alter the metabolism of the cells present in the TME, especially tumor-associated immune cells, which, in turn, interfere with the metabolism of tumor cells. Tumor cells have a high nutrient requirement; however, limited nutrient supply leads to metabolic stress in the TME, which mainly manifests as a hypoxic environment caused by a lack of oxygen and as energy competition caused by a lack of glucose. In addition, metabolites from tumor cells or other cells accumulate to create a low pH environment caused by lactic acid accumulation and a lipid peroxidative environment caused by lipid and ROS accumulation. Therefore, tumor and immune cells undergo metabolic reprogramming to circumvent these limitations, thereby affecting tumor immune response (Figure 3) 165.

The number and composition of immune cells in the TME are heterogeneous. Some tumors are classified as “cold tumors” because of a lack of immune cell infiltration and present an “immune desert.” However, some tumors are classified as "hot tumors" because they are rich in immune cells. Apparently, a “hot tumor” is more responsive to immunotherapy than a “cold tumor” 5. The difference lies in the mechanism by which immune cells find tumor cells. Here, chemokines play an important guiding role, and different subsets of lymphocytes and APCs are recruited to the TME through different chemokine-chemokine receptor signaling pathways 37. In addition, chemokines can directly target non-immune cells including tumor cells and vascular endothelial cells, resulting in differential effects on tumor progression and immunotherapy outcomes 166. Notably, some authors have reported that high-density lipoprotein (HDL) 167 and oxidized lipids 168 can affect the expression of chemokines and their receptors, indicating that lipid metabolism in the TME can affect the expression of chemokines. In turn, some other researchers have shown that chemokines can affect the lipid metabolism of cells; however, the specific mechanism is unknown 169.

### Lipid metabolic reprogramming in cancer-associated fibroblasts

In addition to immune cells, cancer-associated fibroblasts (CAFs) are the most important component of stromal cells in the TME and are the center of cross-communication between various cells. CAFs can secrete several cytokines, chemokines, and extracellular matrix proteins, thereby promoting the occurrence and development of tumors 170. Tumor cells are anabolic, whereas CAFs are catabolic. Some studies have pointed out that their metabolism is coupled, implying that the catabolism of CAFs can provide important metabolic substances for the growth of tumor cells, thus establishing a symbiotic relationship. CAFs demonstrate the reverse "Warburg effect," which is characterized by aerobic glycolysis and leads to lactic acid secretion; the lactic acid is then absorbed and utilized by cancer cells. In addition, CAFs increase the endogenous level of ROS in the TME. In terms of lipid metabolism, CAFs undergo reprogramming, which is manifested as the secretion of lysophosphatidylcholines into the TME, where they exert diverse effects on tumor cells and other cells in the TME 171. On the contrary, CAFs also regulate immune cell-mediated antitumor immunity, including promoting the activation of immunosuppressive cells, enhancing the expression of immune checkpoint molecules, and limiting the function of effector immune cells 172.

### Hypoxic environment

Tumor cells have high oxygen requirements to support their rapid proliferation but the relatively slow and abnormal angiogenesis causes insufficient oxygen supply, which often leads to local hypoxia in tumor tissues. Necrosis, occurring in the hypoxic area, releases tumor antigens to activate the tumor immune response 173. The differences in blood supply to the tumor tissue make hypoxic and oxygen-rich areas coexist and change dynamically, which results in metabolic heterogeneity and the occurrence of a unique phenomenon of "metabolic symbiosis". For example, tumor cells in the hypoxic area away from blood vessels enhance aerobic glycolysis by upregulating GLUT and glycolysis-related enzymes, whereas those in the normal oxygen supply area near blood vessels tend to undergo TCA cycle and OXPHOS to efficiently use glucose and transport saved glucose to the hypoxic area 174.

The functions of some key enzymes of lipid metabolism are affected in a hypoxic environment. For example, SCD1 needs oxygen to catalyze the formation of carbon double bonds to desaturate FAs. Therefore, SFAs are accumulated in the absence of oxygen by preventing the formation of UFAs. Tumor cells enhance the uptake of UFA and its release from LD to restore the SFA/UFA balance. However, hypoxia can also increase the expression of SCD1 through the regulation of SREBP1 175.

HIF-1α and hypoxia-related genes, such as EPAS1, are highly expressed in various hypoxic tumor tissues, which play an important role in tumor angiogenesis, metabolism, and immunity 176. In addition to promoting glycolysis, hypoxia can enhance lipid metabolism through the activation of the PI3K/Akt/mTOR pathway. HIF-1α can inhibit lipid peroxidation for inducing ferroptosis by increasing the expression of FABP3/7, LDLR, and LD to promote lipid uptake and storage 177. Notably, the activation of T cells increases the expression of HIF-1 and Myc, thereby promoting the activity of key enzymes in glycolysis such as GLUT. Hypoxia also induces the persistent activation of dynamin-related protein 1 in NK cells 178, which is involved in the mTOR signaling pathway and leads to mitochondrial fission, thereby decreasing the survival and cytotoxicity of NK cells. In addition, hypoxia can increase the expression of Foxp3, a key transcription factor in Treg cells, which promotes lipid uptake by CD36, OXPHOS, and FAO 179. Meanwhile, hypoxia also promotes the infiltration of Treg cells into tumor tissues. However, HIF-1α can also destroy the stability of Treg cells 180. This contradiction can be attributed to different degrees of hypoxia, implying that mild hypoxia stimulates cell growth, whereas severe hypoxia induces cell death.

### Glucose-deficient environment

Tumor cells are more sensitive to glucose deficiency than other cells in the TME because of their high energy requirements. Tumor-associated immune cells can partially use ketone bodies as alternative energy sources but "glycolysis subtype" tumor cells cannot use ketone bodies instead of glucose 31. Therefore, such tumor cells rely on lipids as the main energy source, which makes them more aggressive and induces immunosuppression.

However, glucose metabolism plays an irreplaceable role in the occurrence and development of tumors. As an important carbon source, intermediates of glycolysis and aerobic oxidation, such as glucose-6-phosphate and pyruvate, can be used as the essential material basis. Specifically, the TCA cycle and PPP provide AcCoA and NADPH for lipid synthesis, respectively. Glycolysis also provides glycerol 3-phosphate for the synthesis of TAG. Compared with FAO and OXPHOS, the rapid energy supply provided by glycolysis can meet the emergency energy needs of tumor cells during proliferation or metastasis. Glycolysis reduces the dependence on oxygen and mitochondrial function, which reduces the production of ETC-derived ROS and inhibits lipid peroxidation. The lactic acid produced by glycolysis can acidify the TME and induce tumor immunosuppression. Hexokinase (HK), a key glycolytic enzyme, inhibits the apoptosis of tumor cells 181, 182.

Therefore, tumor cells compete for glucose and outdo immune cells by upregulating GLUT and other pathways 11. For example, the partial gluconeogenesis pathway is activated in tumor cells, which allows more intermediate flow into the glycolysis pathway to compensate for the lack of glucose. In non-gluconeogenesis tissues, such as lung cancer and colorectal cancer, tumor cells with the expression of PCK1 can use non-sugar energy sources to carry out partial gluconeogenesis because of the lack of downstream key enzymes of gluconeogenesis, such as FBP and glucose-6-phosphatase (G6P). On the contrary, in some cancers, such as liver and kidney cancer, tumor cells expressing key enzymes of gluconeogenesis, including PCK1, FBP, and G6P, can promote the whole gluconeogenesis pathway to produce glucose, which, in turn, inhibits the glycolytic pathway. Notably, FBP can also inhibit PPARα-mediated FAO, thereby inhibiting tumor development 12.

The supply of glucose is equally important for the activation of immune cells, specifically antitumor immune cells. The lack of glucose hinders the activation of T cells, and T cells undergo metabolic reprogramming and shift toward lipid metabolism for survival by activating the AMPK pathway, PPARα signaling pathway, and FAO. In addition, T cells differentiate into Treg cells in response to low glucose levels, which is dependent on the activity of AMPK and PPARα signaling pathways 140. Low glucose levels also inhibit glycolysis induced by mTORC1 signaling, which, in turn, inhibits the production of IFN-γ and granzyme by NK cells 183 and the ability of DCs 152 to activate tumor immune responses.

### Low pH environment

Accumulation of lactic acid produced by glycolysis lowers the pH of the TME. The acidic environment can theoretically lead to apoptosis, thus playing an antitumor role. However, lactic acid transported to the stroma is quickly taken up by tumor cells through MCT1 and utilized in the normal oxygen supply area, thereby maintaining optimal lactic acid levels in the TME for protumor effect. Moreover, metabolic stress of the TME can promote the expression of MCT1, which can also activate the NF-κB signaling pathway 184.

Lactic acid has traditionally been considered a metabolic waste; however, a study has indicated that it can upregulate the expression of vascular endothelial growth factor (VEGF) and HIF-1 185. In addition, tumor cells can use lactic acid as an energy source to generate ATP through a partial TCA cycle, which further strengthens the concept of “metabolic symbiosis”. For example, lactic acid produced by tumor cells in the hypoxic area is excreted into the stroma through MCT4 and then transported to the oxygen-rich area, where tumor cells uptake lactic acid through MCT1 and activate the mTOR signaling pathway. In this process, cells in the oxygen-rich area reduce the consumption of oxygen and glucose, while those in the hypoxic area avoid excessive accumulation of lactic acid. In addition, lactic acid increases the expression of PD-L1 through the lactic acid receptor (GPR81) pathway, eventually leading to tumor immunosuppression 186.

Lactic acid and H^+^ have long been regarded as negative regulators of immunity, but recent studies have indicated that lactic acid can have immune-promoting effects. Lactic acid and H^+^ can inhibit the survival and function of T cells by inhibiting the MPAK signaling pathway 187. In addition, the increased lactic acid uptake by NK cells l decreases intracellular pH, which impairs their energy metabolism and function 188. Lactic acid can promote tumor development by inducing immunosuppression-related cells, such as Treg cells and M2 macrophages 160, which may be related to histone lactonization (Kla). It also enhances the expression of PD-1 in Treg cells 187. In contrast, lactic acid can partially replace glucose as an energy source and increase the stem cell properties of CD8+T cells to enhance antitumor immunity 189. Overall, these observations suggest that the different effects of lactic acid and H^+^ should be distinguished while evaluating the effect of low pH on antitumor responses.

In addition to lactic acid, H^+^ produced by redox reactions occurring in tumor cells can be mainly transported out of the cell via V-ATPases to prevent acidosis and maintain a low pH environment 190. In addition, the CO_2_ produced by intracellular decarboxylation reaction can diffuse to the TME, which results in a low pH environment and affects the survival and function of immune cells 191. A low pH environment can activate lysosomes and proteolytic enzymes to promote the degradation of the extracellular matrix, thereby promoting the invasion and metastasis of the tumor. Therefore, protein carriers such as V-ATPases can be potential targets for cancer therapy 192.

For lipid metabolism, acidic conditions mediate the transport of FA by CD36 and promote the storage of PUFA in LDs, thereby increasing lipid accumulation in tumor cells 184. Acid sensory receptors on the membrane of tumor cells, such as G protein-coupled receptor 1 (OGR1), can sense the low pH and promote the formation of LDs 193. The low pH environment also promotes the lipid peroxidation of ω-3 and ω-6 PUFAs, which increases the toxicity of PUFAs and the sensitivity of tumor cells to ferroptosis. In addition, low pH regulates the expression of SREBP2 target genes, such as HMGCR and ACAT, and increases the biosynthesis of cholesterol 185.

### Lipid peroxidation environment

Most solid tumors are rich in lipids, which are obtained from the surrounding cells or bloodstream. Tumor cells can induce metabolic changes in adjacent adipocytes to release FFA and cholesterol into the TME and then absorb them through FATP 194. The TME is often in a state of oxidative stress. The "respiratory burst" of infiltrating neutrophils and catalytic reactions of LOX, COX, and xanthine oxidase produces a large amount of ROS. Together, they create a lipid peroxidation environment in the TME and produce many "harmful lipids" such as ox-LDL, malondialdehyde (MDA), 4-hydroxynonenoic acid (HNE), and 27-HC 195.

Tumor cells often upregulate lipid uptake and storage to adapt to the low-glucose and high-fat TME. The uptake of ox-LDL by tumor cells promotes the occurrence and progression of tumors because it affects mitosis, regulates the expression of cyclin, and induces the expression of endoplasmic reticulum localization protein (Nogo-B) 196. However, excessive accumulation of intracellular lipids, particularly PUFA, can also lead to lipid peroxidation and ferroptosis. But interestingly, mammary adipocytes can protect triple-negative breast cancer (TNBC) cells from ferroptosis 197.

The high-fat environment has a bidirectional effect on tumor immune response. The high level of FA can activate PPARα signaling in T cells, which, in turn, promotes FAO and OXPHOS; however, the depletion of FA in T cells hinders their proliferation and antitumor effect 198. Likewise, the activation of PD-1 signaling inhibited the TCR and CD28-mediated PI3K/AKT/mTOR pathway to promote FAO 199. In addition, high levels of cholesterol can enhance the number and antitumor effect of NK cells by upregulating LDLR 149. In contrast, excessive FAs, including oxidized lipids, inhibit the survival and antitumor effect of T cells. Specifically, T cells take up ox-LDL and AA via CD36, leading to intracellular lipid peroxidation 74. Similarly, high levels of cholesterol can induce endoplasmic reticulum stress in T cells and upregulate PD-1 expression 200. However, an increase in the intracellular cholesterol level by inhibiting ACAT1 can promote the activation of T cells 201. This contradictory result may be attributed to the distribution of intracellular cholesterol, implying that inhibition of cholesterol storage may increase the flow of free cholesterol to the cell membrane, which promotes the formation of lipid rafts and the aggregation of TCR. Overall, these observations suggest that lowering the level of cholesterol in the TME is not necessarily beneficial.

### Tumor microenvironment induces autophagy

Autophagy is considered a self-protective mechanism of cells. In the TME, metabolic stress, oxidative stress, and immune signals can induce autophagy by increasing the activity of autophagy-related genes and the ability of intracellular lysosome transport, which allows for efficient degradation of cellular components. Autophagy is regulated by two antagonistic regulatory kinases, AMPK (activator) and mTORC1 (inhibitor) 113.

Tumor cells can achieve energy recycling and substance renewal through autophagy, which increases the lipid pool and helps tumor cells adapt to the nutrient competition 202. In addition, autophagy can reduce intracellular oxidative stress and inflammation and prevent DNA damage, thereby reducing lipid peroxidation. Notably, the resistance of tumor cells to chemotherapeutic and targeted drugs is partly caused by abnormal activation of autophagy 203. However, the use of autophagy inhibitors alone has not achieved ideal antitumor effects in clinical trials. Therefore, autophagy inhibitors are used in combination with chemotherapy or immunotherapy 204. In contrast, excessive autophagy induces apoptosis and can promote ferroptosis in tumor cells. Although autophagy can scavenge peroxides, the phagocytosis of ferritin releases large amounts of Fe^2+^ and increases the intracellular labile iron pool. In addition, mtDNA stress triggered autophagy-dependent ferroptosis 205.

Autophagy also affects the tumor immune response. Autophagy can selectively degrade MHC I molecules, thereby reducing the immunogenicity of tumor cells. In contrast, the inhibition of autophagy in tumor cells can increase the expression of tumor surface antigens. Autophagy in MDSCs degrades MHC II molecules and inhibits the activation of T cells 206. However, autophagy of immune cells can support their adaptive survival in the TME 207.

Overall, autophagy is a double-edged sword for tumor and immune cells. Inhibition of autophagy promotes antitumor immune responses to a certain extent and represents a novel direction for cancer immunotherapy 208.

## Abnormal cell death related to lipid peroxidation and tumor immunity

**Figure 4:**
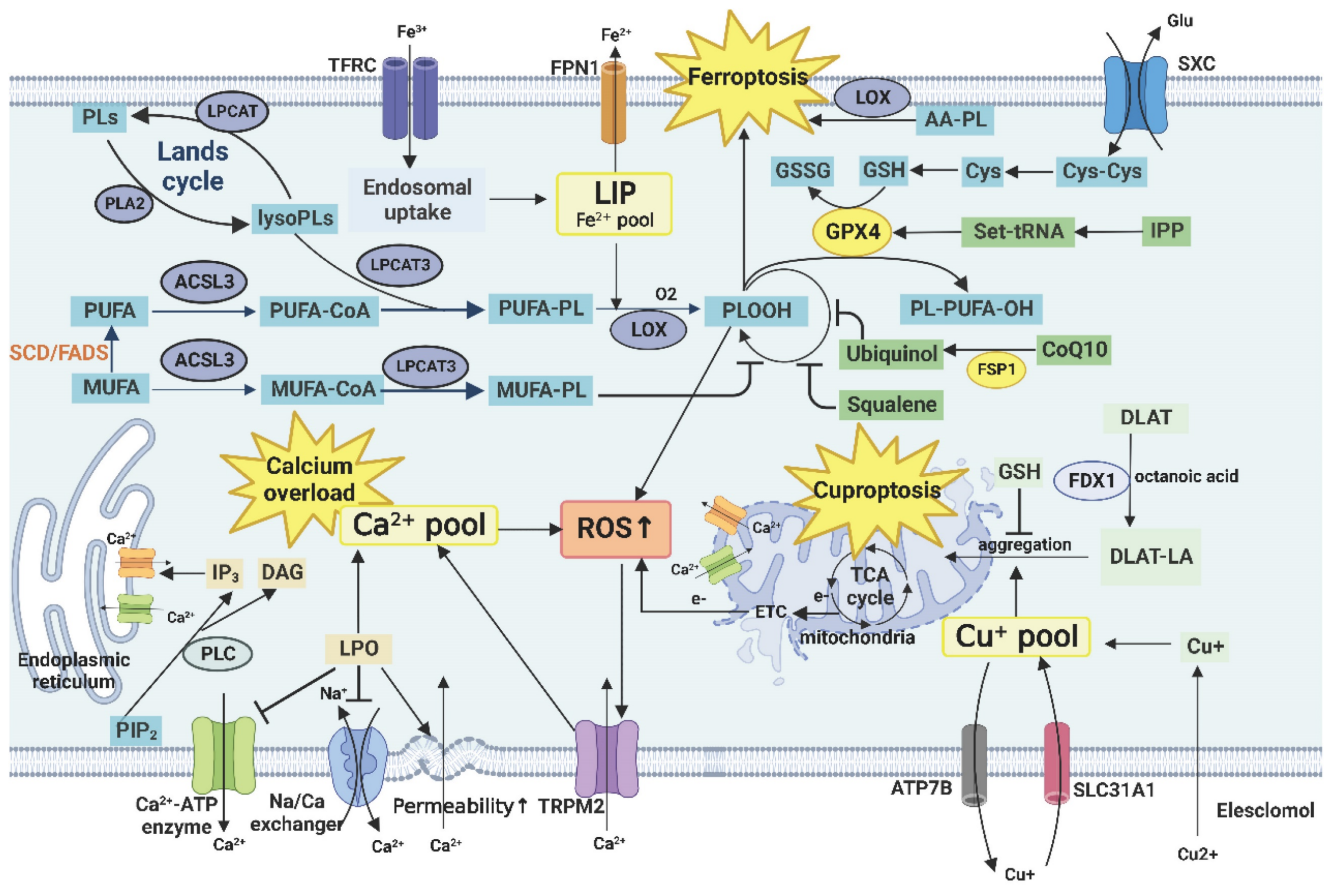
** Lipid peroxidation-related cell death.** The cell death modes related to lipid peroxidation mainly include ferroptosis, cuproptosis, and calcium overload. Notably, intermediates in the synthesis of TC can play a protective role against ferroptosis. In ferroptosis, ACSL4 is involved in the attachment of PUFAs to coenzyme A, and LPCAT3 promotes the esterification and incorporation of PUFA-CoA into the membrane phospholipids. The resulting PUFA-PL, specifically AA-PL and AdA-PL, are major peroxidation substrates for ferroptosis. PUFA-PL are readily oxidized by ROS (specifically HO•) to generate PLOOH, which damages the structure of membrane lipids if not effectively neutralized. The peroxidation could be initiated in cells both enzymatically and nonenzymatically. Nonenzymatic peroxidation of lipids is mostly catalyzed by LIP. Enzymatic peroxidation is driven by multiple LOX and POR. PL with AA-PL is also susceptible to LOX-mediated radical oxidation to PLOOH and 5-HPETE. SXC/GSH/GPX4 axis and FSP1/CoQ10/NADPH axis are two major monitoring systems to inhibit PL peroxidation and prevent ferroptosis. In cuproptosis, excessive Cu^2+^ directly binds to the lipoacylated component of the TCA cycle, such as dihydrolipoic acid transacetylase (DLAT), and results in the abnormal aggregation of insoluble DLAT and subsequent downregulation of Fe-S cluster proteins. In calcium overload, lipid peroxidation increases the permeability of the membrane. ROS can activate TRPM2 and cause massive Ca^2+^ influx. Then, the excessive intracellular Ca^2+^ further increases the Ca^2+^ uptake by mitochondria, which leads to the formation of calcium phosphate deposits and inhibits the synthesis of ATP, thereby decreasing the activity of the Ca^2+^ pump in the endoplasmic reticulum and cell membrane.

Lipid peroxidation is the process by which ROS reacts with PL, enzymes, membrane receptors, and other PUFA side chains to form lipid peroxidation products (LPO) such as MDA and HNE. It eventually destroys membranes and important cellular components such as proteins and nucleic acids 209. Cell death related to lipid peroxidation involves ferroptosis, cuproptosis, and calcium overload. Tumor cell death can promote the infiltration of immune cells and activate antitumor immune responses through the expression of damage-related molecular patterns such as calreticulin and the release of immune effectors, such as tumor antigens, high mobility group box 1, ATP, and heat shock proteins (HSP70 and HSP90). This process is also called tumor immunogenic cell death (Figure 4) 210.

### Lipid peroxidation in tumor cells

Tumor cells are more susceptible to lipid peroxidation than normal cells. The production rate of ROS is higher in tumor cells. ETC is prone to leak out ROS during OXPHOS because of the defects in intracellular mitochondria and the dependence of energy metabolism on them. In addition, intracellular NADPH oxidase can produce superoxide, which is rapidly converted to H_2_O2 by superoxide dismutase (SOD). Moreover, the clearance rate of ROS is lower in tumor cells. Unlike normal cells, tumor cells have low levels of antioxidant enzymes and their antioxidant barrier is defective. Therefore, tumor cells inhibit lipid peroxidation by reducing mitochondrial activity (Warburg effect), increasing the production of PGs, and reducing the level of PUFA in the membrane. Low levels of ROS are often maintained in tumor stem cells and tumor cells in the proliferative state or with drug resistance 209, 211.

However, long-term maintenance of moderate levels of ROS is beneficial for tumor progression. ROS-induced DNA damage leads to genomic instability, which drives the accumulation of oncogenic mutations. A certain level of ROS and LPO act as secondary messengers to activate related transcription factors and participate in the regulation of signaling pathways, such as PI3k/Akt, RTK/Ras, VEGF, NF-κB, and COX-2, to promote tumor inflammation and immune response 212. In addition, lipid peroxidation induces non-enzymatic oxidative modification of proteins and makes them immunogenic 209.

Taken together, the bidirectional effect of lipid peroxidation on tumors suggests that different types and stages of tumors and immune responses have different requirements for the expression of ROS and LPO; therefore, tumor immunotherapy targeting lipid peroxidation should be carefully designed 213.

### Ferroptosis

Ferroptosis, a newly emerged form of non-apoptotic regulatory cell death caused by LPO overload in the membrane, is considered a by-product of abnormal lipid metabolic reprogramming. The balance between ferroptosis-related peroxidation and antioxidation systems determines whether the cell undergoes apoptosis or not 214. Activated CD8+T cells accelerate the ferroptosis of tumor cells by hypersecreting IFN-γ during anti-PD-L1 immunotherapy 215. Ferroptosis inhibition in tumor cells is also one of the resistance mechanisms of PD-1/PD-L1 inhibitors. In addition, both radiotherapy and targeted therapy with TYRO3 receptor tyrosine kinase inhibitors promote ferroptosis in tumor cells, and exert excellent tumor-control effects in combination with immunotherapy 216.

Oxygen and iron are essential factors in the ferroptosis-related peroxidation system that drive lipid peroxidation to produce phospholipid peroxides (PLOOH), which can damage the structure of membrane lipids if not effectively neutralized. ACSL4 converts PUFAs into their respective acyl-CoA, while LPCAT3 promotes the esterification and incorporation of PUFA-CoA into membrane PLs 217. The resulting PUFA-PLs, specifically AA-PL and adrenocorticotropic acid (AdA)-PL, are the major peroxidation substrates for ferroptosis, which are easily oxidized by ROS to produce PLOOH 218. The peroxidation could be initiated enzymatically by multiple LOXs and P450 oxidoreductases and nonenzymatically by LIP 219. CD8+T cell-derived IFN-γ stimulates ACSL4 and increases the incorporation of AA into C16 and C18 acyl chain-containing phospholipids. Therefore, IFN-γ combined with AA has become one of the means to control immunogenic tumors 220.

The System X-(SXC)/GSH /GPX4 and FSP1/CoQ10/NADPH axes are two major monitoring systems in the ferroptosis-related antioxidant defense system to inhibit PL peroxidation and prevent ferroptosis. SXC, GPX4, and FSP1 are highly expressed in tumors and are related to the poor prognosis of patients, suggesting that tumor cells tend to inhibit their ferroptosis 221. IFN-γ derived from immunotherapy-activated CD8+T cells and the radiotherapy-activated ataxia telangiectasia mutated gene synergistically repress SLC7A11, and this combination strongly promotes lipid oxidation and ferroptosis of tumor cells. A decreased expression of SLC3A2 and an increase in IFN-γ and CD8+T cells are associated with clinical benefits 222. The androgen receptor promotes GPX4 overexpression in the luminal androgen receptor subtype of TNBC, which assists tumor cells in escaping ferroptosis 223.

Notably, ferroptosis may also occur in immune cells, and this may be a specific mechanism of tumor immunosuppression. For example, CD36-mediated uptake of FA by CD8+T cells in the TME can induce lipid peroxidation and ferroptosis, which reduce the production of cytotoxic cytokines and decreases their antitumor ability 224. GPX4-deficient T cells can rapidly accumulate membrane LPO and lead to ferroptosis, and therapeutic induction of ferroptosis in tumor cells by GPX4 inhibitors may have unnecessary effects on T cells. ACSL4 is also essential for the ferroptosis and the maintenance of immune function of CD8+T cells 225. Knockout of GPX4 promotes the occurrence of ferroptosis in Treg cells, inhibits the immune escape function, and enhances the response of TH17 cells by secreting IL-1β to promote the antitumor immune function 226.

### Cuproptosis

Cu^2+^ is a necessary cofactor for enzymes that mediate basic cellular functions, including mitochondrial respiration and redox reactions. Although Cu^2+^ does not participate directly in the mitochondrial respiratory chain, it promotes the activity of key enzymes in the TCA cycle and inhibits the leakage of ROS from the respiratory chain. Therefore, compared with normal cells, tumor cells need higher levels of Cu^2+^ to maintain mitochondrial metabolic activity and intracellular redox balance. The level of Cu^2+^ in serum or tumor cells of tumor patients is higher than that in normal individuals, which can induce cuproptosis in cells 227.

Excessive Cu^2+^ can directly bind to the lipoacylated components of the TCA cycle, such as dihydrolipoic acid transacetylase (DLAT), and result in the abnormal aggregation of insoluble DLAT and subsequent downregulation of Fe-S cluster proteins, eventually leading to proteotoxic stress and ultimately cell death. Therefore, cells dependent on mitochondrial respiration are more sensitive to Cu^2+^ ions than cells that rely on glycolysis 228. Therefore, tumor cells reduce the activity of mitochondria through the Warburg effect to avoid cuproptosis 229. In addition, Cu^2+^ promotes the accumulation of ROS and induces lipid peroxidation. Cu^2+^ can also directly activate various angiogenic factors, including VEGF, fibroblast growth factor 2, TNF-α, and IL-1, thereby promoting tumor angiogenesis. Cu^2+^ regulates autophagy through ULK1 and ULK2 (serine/threonine kinases). High levels of Cu^2+^ are associated with an increased expression of PD-L1, which may help tumors resist immunotherapy 230. Therefore, several researchers have analyzed the relationship between Cu^2+^ and tumor-associated immune cells and established models to predict the effect of Cu^2+^ on tumor immunotherapy.

### Calcium overload

Mitochondria, as an intracellular calcium pool, are involved in the regulation of intracellular calcium homeostasis. Lipid peroxidation increases the permeability of the membrane under oxidative stress. ROS activate TRPM2 (H_2_O_2_ -dependent Ca^2+^ channel) on the surface of tumor cells in the TME leading to massive Ca^2+^ influx. The excessive intracellular Ca^2+^ further increases the uptake of Ca^2+^ by mitochondria, which leads to the formation of calcium phosphate deposits and inhibits ATP synthesis. Consequently, the activity of the Ca^2+^ pump in the endoplasmic reticulum and cell membrane further decreases, which, in turn, increases intracellular Ca^2+^ levels. Then, excessive Ca^2+^ can abnormally activate various Ca^2+^-dependent degradation enzymes. For example, the phospholipase activation promotes the hydrolysis of PLs, resulting in membrane destruction 231.

However, cell migration and invasion in some tumors, such as glioblastoma and esophageal squamous cell carcinoma (ESCC), are critically dependent on Ca^2+^ signaling 232. The hyperactive intracellular calcium oscillations caused by the Ca^2+^ influx in the endoplasmic reticulum from the cytoplasm or the excessive opening of some store-operated Ca^2+^ entry (SOCE) channels are associated with the proliferation of tumor cells. Notably, extracellular zinc exerted rapid inhibitory effects on SOCE and intracellular Ca2+ oscillations in the ESCC cells, thereby suggesting a possible molecular basis for zinc-induced cancer prevention 233.

Calcium overload can activate the GSDME protein, which induces pyroptosis in tumor cells and sensitizes immunotherapy. Currently, several methods, such as Ca^2+^ nanoparticles and calcium hydride (CaH_2_) administration, are being evaluated to induce calcium overload in tumor cells to assist immunotherapy and develop a new tumor treatment strategy, namely ion interference therapy 234, 235.

## Clinical applications of lipid metabolic reprogramming

Targeting lipid metabolism is more sensitive and beneficial than targeting glucose metabolism for tumor immunotherapy. Tumors with metabolic heterogeneity are more adaptable to interventions in glucose metabolism. Further, drugs that target glucose metabolism can also inhibit antitumor immune cells because of their dependence on glucose. In contrast, protumor immune cells are mainly dependent on lipid metabolism and tumor cells are more sensitive to lipids than glucose. In addition, existing drugs for disorders of lipid metabolism and NSAIDs can also be reused to treat abnormal lipid metabolism in the TME 236. Ferroptosis and autophagy are also linked to lipid metabolism, which can further improve the effect of immunotherapy (Table 3).

However, although lipid metabolic reprogramming leads to an immunosuppressive TME, lipid remains the important energy and material basis of the cells for antitumor immune response. Therefore, therapies targeting lipid metabolism should be specific and targeted to certain components and pathways of lipid metabolism in different types and stages of tumor and immune cells to maximize their sensitizing effects on immunotherapy 237. Presently, most lipid-related antitumor drugs are still in preclinical studies and have yet not achieved satisfactory therapeutic effects or even have contradictory results (especially when used alone). Nevertheless, the research on such drugs mainly provides new possibilities for sensitizing tumor immunotherapy and their limitations prompt us to further elucidate the associated mechanisms 60.

### Targeting fatty acid metabolism

#### Interventions in the uptake of fatty acids

CD36 inhibitors hinder tumor development, specifically metastasis and angiogenesis in the TME 238 and have shown synergistic effects in combination with the FASN inhibitors and anti-PD-1 therapy 239. CD36 inhibitors can not only enhance the antigen presentation ability of DC but also reduce the number of Treg cells and upregulate PD-1 by inhibiting the PPAR pathway, thereby promoting the survival and function of CD8+T cells. CD36 inhibitors are related to the inhibition of lipid peroxidation and ferroptosis in immune cells 224.

In addition, the potential protumor role of the FABP has been demonstrated. The inhibition of FATP reduces lipid uptake, invasion, and growth in tumors such as melanoma 240, 241. Notably, FATP2 can promote the utilization of AA and the synthesis of PGE2 in MDSCs, thereby mediating immunosuppression 242. Therefore, FATP inhibitors can also improve the effect of immunotherapy.

#### Interventions in the synthesis of fatty acids

The development of tumor cells and immune cells is affected by the expression of ACLY and its product AcCoA. Inhibition of ACLY can reduce the levels of intracellular FA and cholesterol and inhibit upstream glucose metabolism 243. Notably, ACLY is involved in the IL-2-mediated activation of Treg, CD4+T, and CD8+T cells 244, 245. However, the *in vivo* efficacy of existing ACLY inhibitors in tumor models (specifically on tumor immune response) has not been definitive; however, the recently identified 3D structure of ACLY holoenzyme tetramer is expected to provide new insights into the mechanism of ACLY inhibition leading to the development of effective ACLY inhibitors 246.

The pathway of AcCoA synthesis by acetyl-CoA synthetase (ACSS) can bypass the inhibition of ACLY, which makes tumor cells resistant to ACLY inhibitors. Therefore, ACSS2 inhibitors not only hinder the growth of acetate-dependent tumors but also improve the antitumor effect of ACLY inhibitors. In addition, ACSS2 inhibitors promote antitumor immune responses. The expression of ACSS2 in cervical squamous cell carcinoma is proportional to that of PD-L1 and is associated with the infiltration of B cells, CD4+T cells, CD8+T cells, and CAFs 247.

ACC inhibitors can impede the growth of the tumor and enhance the efficacy of chemotherapy and targeted drugs 248. However, inhibition of ACC increases the expression of SREBP1 and leads to the accumulation of TAGs in blood. Notably, diacylglycerol acyltransferase 2 (DGAT2) inhibitors can downregulate the activity of SREBP1 and inhibit the expression of genes related to lipid synthesis. Therefore, the combination of ACC and DGAT2 inhibitors can improve the antitumor effect and reduce the side effects, such as hyperlipidemia, of ACC treatment 249. Given that AMPK is a key inhibitor of ACC, a classical agonist of AMPK metformin can block lipid metabolism in tumor cells. Metformin has an important role in tumor immunomodulation. For example, metformin can promote the transformation within the TME to an antitumor response by increasing the number of CD8+T cells and decreasing that of M2 macrophages 250.

FASN inhibitors inhibit the energy supply of tumor cells and disrupt their membrane composition. Specifically, FASN inhibitors can induce ferroptosis in tumor cells by reducing the levels of intracellular TAG and PC and increasing the levels of PUFA in the membrane through the Lands cycle. In addition, FASN inhibitors promote the formation of lipid rafts and the activation of the TLR4 signaling pathway 251. Notably, FASN inhibitors markedly reduce the release of ROS, IL-10, and TNF-α by M2 macrophages and inhibit the activation of Treg cells. FASN inhibitors also prevent tumor metastasis after the withdrawal of antiangiogenic therapy 252. Several FASN inhibitors have shown fewer side effects and better antitumor effects in preclinical studies; however, none of them has yet been approved for clinical use.

#### Interventions in the desaturation of fatty acids

Currently, SCD1 inhibitors are in the preclinical stage, and the existence of SCD1 compensatory or alternative pathways can easily make tumor cells resistant to SCD1 inhibitors. The expression of the transcription factor FOSB, which regulates the activity of SCD1, rapidly restores the SCD1 level and induces drug resistance 253. Tumor cells with high expression of FADS1/2 can bypass the effect of SCD1 inhibition 254. Therefore, a combination of SCD1 and FADS inhibitors effectively blocks the proliferation of tumor cells. Notably, SCD1 inhibitors have also demonstrated synergistic antitumor effects with targeted therapies. These inhibitors can increase the infiltration and activation of DC and CD8+T cells and enhance the efficacy of anti-PD-1 therapy 60.

#### Interventions in the lipolysis of triglycerides

The expression of monoacylglycerol lipase (MAGL) is markedly elevated in many tumor cells, which leads to an increase in intracellular FFA levels that are closely related to cancer-associated cachexia 255. Through the MAGL-FFA pathway, tumor cells can promote the production of lipid signaling molecules such as LPA, LPC, and PGE2, which promote tumor development and affect tumor immune response 256. Therefore, in preclinical studies, MAGL inhibitors have reduced the invasiveness of tumors and improved the efficacy of tumor immunotherapy.

#### Interventions in the decomposition of fatty acids

The drug-resistant tumor cells, such as TNBC, exhibit dependence on FAO 257; therefore, CPT1 inhibitors can synergistically enhance the antitumor effects of antiangiogenic, chemotherapy, and immune drugs. CPT1 inhibitors can eliminate the protumor effect of TAMs, inhibit the infiltration of MDSCs, and enhance the antitumor function of T cells 258. Further, CPT1 expression can be used as a potential marker of tumor immunosuppression.

### Targeting the prostaglandin pathway

COX-2 inhibitors are effective in the prevention and treatment of tumors (specifically gastrointestinal tumors) and prostate cancer 259. However, they are beneficial only when combined with chemotherapy drugs or immune checkpoint inhibitors (ICIs). Notably, the EGFR signaling pathway reduces the expression of intracellular 15-PGDH; therefore, the combined blocking effect of EGFR and COX-2 may also enhance the antitumor effect of COX-2 inhibitors 260. In addition, the expression of indoleamine 2, 3-dioxygenase 1, an important immunomodulatory enzyme in tumor cells, is related to the activation of the COX2/PGE2 axis; therefore, COX-2 inhibitors can enhance the effect of the anti-PD-1 therapy to some extent 261.

The mechanism of COX-2 inhibitors is not entirely dependent on the role of COX-2 but is related to COX-1 or even independent of the PGE2 pathway. COX-1 is highly expressed (even more than COX-2) in ovarian and cervical cancers. Therefore, COX-1 inhibitors are more effective in treating these COX-1-dominated tumors 262. However, both COX-1 and COX-2 inhibitors may increase the risk of cardiovascular diseases and gastrointestinal mucosal injury in patients. Therefore, it is necessary to further determine the indications for COX inhibitors or develop specific inhibitors against downstream targets of COX, such as PGES and prostaglandin receptors to reduce their side effects and achieve better efficacy. For example, mPGES-1 inhibitors reduce PGE2 while avoiding adverse reactions such as cardiovascular diseases, and they even demonstrate antithrombotic and antiatherosclerotic effects 259.

### Targeting cholesterol metabolism

#### Interventions in the uptake of cholesterol

LDLR is upregulated through EGFR/PI3K/AKT/SREBP-1 signaling, which enhances the uptake of LDL-C and plays an important role in the growth of cancers such as lung cancer and breast cancer 136. In addition, LDLR is involved in the regulation of tumor immunity. It can interact with the TCR complex and regulate its recycling and signaling, thus enhancing the cytotoxic effect of CD8+T cells. Therefore, LDLR inhibitors can enhance the efficacy of tumor immunotherapy. For example, PCSK9, as a regulator of cholesterol metabolism, can not only reduce the level of cholesterol in blood but also negatively regulate the expression of LDLR, thereby synergistically enhancing its antitumor effect with PD-1 inhibitors 63.

#### Interventions in the synthesis of cholesterol

Statins, in addition to lowering blood lipids, can inhibit the activity of HMGCR. They can remodel the cytoskeleton and lipid rafts by reducing cholesterol in the membrane, thereby inhibiting the proliferation and metastasis of the tumor 263. In addition, statins can downregulate the TNF-α receptor and inhibit the NF-κB signaling pathway to induce apoptosis of tumor cells. Although the protective effect of the MVA pathway on ferroptosis is weakened, statins can exert obvious antioxidant effects through other pathways, including PI3K/Akt, AMPK, Rho/ROCK, and NO signaling pathways, and reduce ROS-induced tumorigenesis 264. Further, statins block the stabilizing effects of cholesterol on PD-L1 and PD-1, which are expressed on T, NKT, and Treg cells. Statins can also reduce the level of PD-L1 by inhibiting AKT and β-catenin-mediated signaling pathways and the expression of long non-coding RNA (SNHG29) 265. Similarly, inhibition of SREBP signaling also downregulates the expression of PD-1 on Treg cells 156.

Of note, fat-soluble statins have better antitumor effects than water-soluble statins but the mechanism is unknown 266. However, some clinical trials of statins have not achieved ideal effects, possibly because the doses used are similar to those used for lipid-lowering therapy and fail to achieve sufficient concentrations. Similarly, some studies have reported that after removing the effect of cholesterol, statins may not affect the prevention and prognosis of some cancers, especially liver cancer, and even promote their development. This may be attributed to statin-mediated activation of the SREBP signaling pathway by negative feedback, thus resisting or even reversing the effect of statins on lowering cholesterol 267. Therefore, the combined therapy with SREBPs inhibitors and statins may have improved efficacy. In addition, p53 mutation can be used as a marker to reflect the sensitivity of statins; however, the use of statins worsens the prognosis of patients with wild-type p53 130.

Squalene synthase (SQS) and squalene epoxidase (SQLE) also affect the synthesis of cholesterol and tumor immune response. SQS inhibitors not only interfere with the squalene synthesis downstream but also increase the CoQ10 synthesis upstream, which may diminish their antitumor effect 268. Similarly, SQLE was upregulated in pancreatic adenocarcinoma, and its levels were significantly correlated with tumor-associated immune cells, immune checkpoints, and biomarkers of the TME. Specifically, the expression of SQLE was negatively correlated with CD4+T cells but positively correlated with CD8+T cells and neutrophils 269. Therefore, SQLE can be used as a potential marker of tumor immune response.

#### Interventions in the storage of cholesterol

The high expression of ACAT1 in tumor-associated immune cells can reduce the level of intracellular free cholesterol and promote the composition and function of the membrane. For example, ACAT1 inhibitors can enhance the cholesterol level in the membranes of CD8+T cells and promote the formation of immune synapses, which enhances immune recognition and cytotoxic effects of CD8+T cells 270. Therefore, ACAT1 can be used as a marker to predict tumor immune response to a certain extent.

### Targeting pathways regulating lipid metabolism

In contrast to the key enzymes and receptors specific for lipid metabolism, targeting the upstream regulatory pathways is more complicated because such pathways are also involved in regulating other cellular processes, such as glucose metabolism, amino acid metabolism, and autophagy. The drugs targeting the regulation of lipid metabolism fail to achieve ideal therapeutic effects and show various side effects or even the paradoxical phenomenon that both forward and reverse regulation of the same signaling pathway will produce antitumor effects. Therefore, underlying mechanisms need to be further studied 248.

#### Interventions in the AMPK and mTOR signaling pathways

Presently, some AMPK agonists, such as metformin, have been reported to have good antitumor effects and are being tested in clinical trials 250. However, AMPK has a bidirectional effect on tumors and appears to promote the survival of tumor cells under the metabolic stress of the TME. Therefore, attempts are being made to develop AMPK agonists that target specific types and stages of tumors 112. Furthermore, the AMPK targeting therapeutic strategies should shift from simple agonists to partial inhibitors in certain tumors to achieve the best efficacy.

AMPK pathways act through mTORC1 inhibition; therefore, inhibition of mTOR signaling has been explored as an antitumor strategy but the results were not optimal 271. Rapamycin analogs cannot completely inhibit the phosphorylation of mTORC1 effector 4EBP and instead lead to compensatory upregulation of mTORC2/AKT activity. Since PI3K/Akt mediates the main pathway of mTOR, a new generation of dual PI3K/mTOR inhibitors are currently being evaluated preclinically, which have achieved better antitumor effects 272. The survival and function of tumor-associated immune cells require mTOR. Therefore, mTOR inhibitors not only hinder the immunosuppressive function of Treg cells but also hinder the antitumor effect of CD8+T and NKT cells. This implies that mTOR inhibitors inhibit antitumor immunity even while positively promoting the formation of immunosuppressive TME. Furthermore, mTOR inhibitors may increase the possibility of developing autoimmunity in the long term because of the suppression of Treg cells 273. Therefore, attempts can be made to develop mTOR inhibitors that target specific immune cells.

#### Interventions in PPAR signaling pathway

The function and expression of PPARs are heterogeneous in different types and stages of tumors. PPARα/γ agonists exert antitumor effects by lowering blood lipids and promoting FAO. In addition, PPARγ agonists have been found to inhibit tumor proliferation, invasion, and angiogenesis. PPARγ agonists can also enhance the function of CD8+T cells and promote the effectiveness of anti-PD-1 therapy 274. Interestingly, PPARγ inhibitors are effective in the treatment of some cancers, such as liver, colorectal, and gastric cancers. PPARα inhibitor can inhibit the proliferation of tumor cells by regulating CPT1 (essential for fatty acid metabolism and energy production). In addition, PPARα inhibitors were demonstrated to eliminate the TDE-induced dysfunction of DC immune function 275. Overall, agonists are effective in treating tumors with low expression of PPARs, whereas inhibitors are effective in treating tumors with high expression of PPARs.

#### Interventions in SREBP and LXR signaling pathways

SREBP1 inhibitors can significantly inhibit the growth of tumor cells. Currently, most of the SREBP1 inhibitors are in the preclinical evaluation stage. SREBP1 inhibitors can enhance the effect of radiotherapy, anti-PD-1 therapy, and polyadenosine diphosphate ribose polymerase inhibitors. SREBP1 inhibitors have also been found to reverse the immunosuppressive effect of Treg cells and reduce the protumor effect of M2 macrophages 276.

LXR agonists can cause the depletion of cholesterol in tumor cells and thus reduce lipid raft signaling. In addition, LXR agonists can induce caspase-dependent apoptosis and block survival signals such as EGFR and Akt 136. LXR agonists can inhibit the survival and immunosuppressive effect of MDSCs while promoting the differentiation of M1 macrophages, resulting in an effective antitumor response 277. Therefore, LXR agonists combined with ICIs may demonstrate an enhanced antitumor effect. In addition, the activation of LXRs inhibited PPARα and then inhibited the expression of FAO-related genes. Therefore, a combination of LXR agonist and PPARα inhibitor can synergistically enhance tumor growth inhibition 278.

### Targeting ketone body metabolism

KD therapy activates ketogenesis in the body through a high-fat low-carbohydrate diet, thereby increasing the concentration of ketone bodies in the blood to replace glucose as the main energy source. This therapy inhibits "glycolysis subtype" tumor cells but is less effective against immune cells. Furthermore, KD therapy can improve the ability of immune cells to resist oxidative stress and thus promote antitumor immunity 279. In addition, KD therapy regulates p53 and mTOR signaling pathways, controls inflammatory responses, and inhibits tumor angiogenesis 280. Notably, KD therapy involves high-fat intake but it does not cause disorders of lipid metabolism such as hyperlipidemia (which is related to the high intake of ω-3PUFA). KD includes "good lipids" such as PUFA, whereas HFD includes "bad lipids" such as SFA and cholesterol 62. However, KD therapy has a low compliance rate. A study has pointed out that BHB is the key to the antitumor effect of KD therapy. BHB supplementation to the level of KD therapy can achieve a similar antitumor effect and enhance the efficacy of ICIs and can solve the problem of poor compliance and enhance the efficacy of tumor immunotherapy. However, the role of BHB in tumors is still unclear, and a study even indicates that BHB can promote the development of some tumors 281.

### Targeting ferroptosis

#### Interventions in phospholipid unsaturation

In addition to directly targeting tumor cells, CD8+T cells can also promote the ferroptosis of tumor cells. T-cell-derived IFN-γ combines with arachidonic acid to activate ACSL4, and the activated enzyme increases the levels of PUFA in the cell membrane, thereby changing the lipid distribution pattern of tumor cell membranes. These changes increase the susceptibility of membranes to lipid peroxidation and naturally induce ferroptosis of tumor cells. However, ACSL4 is the key enzyme of lipid metabolism, which mediates the remodeling of GPLs and the activation of FAs 282. ACSL4 is upregulated in some tumor cells and is related to the invasion of tumors, such as breast and colon tumors 283. Therefore, cancers can be of two types according to the role of ACSL4 in promoting lipid metabolism and ferroptosis. The first type of cancers has ferroptosis dominance patterns such as lung 284, ER+ breast 285, and cervical cancers 286. A study has suggested that targeting the ACSL4 pathway is a potential anticancer strategy; however, this has not yet been confirmed. The second type of cancers have lipid metabolism dominance patterns including ER- breast cancer 287, HCC 288, CRC 289, and prostate cancer 287. ACSL4 inhibitors, such as triacsin C, thiazolidinediones, and troglitazone, have a potent antitumor effect in these malignant tumors 290.

LPCAT3 further increases the ferroptosis sensitivity of tumor cells. LPCAT3 knockout reduced the composition of PUFA-PL in tumor cells, thereby inhibiting ferroptosis and promoting tumor progression 291. In addition, LPCAT3 limited ER stress induced by lipid overload 292. In macrophages, LXR-mediated activation of LPCAT3 promotes antitumor immunity. Although the activation of LPCAT3 can inhibit tumor progression, there are currently no antitumor drugs that target LPCAT3, and further research is needed to determine the mechanism of action by which LPCAT3 promotes antitumor immunity and develop LPCAT3-targeted therapies 293.

#### Balance of regulation between peroxidation and antioxidation

Iron-catalyzed oxidation and enzymatic oxidation ultimately lead to ferroptosis and are the two targets in the context of current research on ferroptosis-targeting therapies. The growth of tumor cells (especially tumor stem cells) depends on the role of iron, and high iron intake increases the risk of tumors (such as HCC and breast cancer) 294. Increasing iron absorption 295, reducing iron storage 296, or restricting iron outflow will lead to increased iron accumulation and metabolism, thus directly or indirectly enhancing LPO. Salinomycin, an antibacterial drug, actively triggers the degradation of ferritin in lysosomes, leading to further iron loading in organelles, thus activating ferroptosis 297. Neratinib and lapatinib increase the expression of transferrin, which is responsible for iron transfer into cells, and decrease the expression of ferroportin, which is responsible for clearing iron from cells. Therefore, these drugs may induce ferroptosis by modulating iron metabolism in the cells 298, 299.

GPX4 and SXC are key enzymes that inhibit ferroptosis. Knockout of GXP4 or inhibition of SXC leads to the accumulation of intracellular LPO, resulting in ferroptosis of tumor cells. Many antineoplastic drugs targeting GPX4 and SXC are also being studied or used clinically; these drugs include SLC7A11 inhibitors (sorafenib and sulfasalazine) 300, 301, GPX4 inhibitors (altretamine and withaferin A) 302, 303, and GCL inhibitors (sulfoximine) 304. Combining GPX4 inhibitors with immunotherapy can further activate T cells in the TME, subsequently enhancing their killing effect on tumor cells. Cyst(e)inase, an engineered enzyme that degrades cystine and cysteine, could be combined with a PD-L1 blocker to significantly improve LPO in tumor cells 305.

#### Targeting ferroptosis of immune cells

Targeting ferroptosis in tumor cells may exert unintended effects on CD8+T cells in the TME. CD8+T cells are more sensitive to ferroptosis induced by GPX4 inhibitors, and overexpression of FSP1 induces ferroptosis resistance and maintains CD8+T cell function (antitumor activity) in the TME. Therefore, FSP1 may have potential therapeutic applications in cancer immunotherapy. Promoting the ferroptosis of immunosuppressive cells, such as Treg cells, M2 macrophages, and MDSC, is a potential antitumor strategy and ferroptosis-targeting drugs may emerge in the future 306.

### Lipids as drug carriers

The therapies targeting lipid metabolism exploit the biological characteristics of lipids, whereas the physical and chemical properties of lipids, such as liposolubility and polarity, determine their use as effective drug carriers, including exosomes and liposomes.

Currently, the immunotherapy strategies that target exosomes mainly inhibit the production of TDEs or interfere with the exosomes released from immune cells. GW4869 is an inhibitor of neutral sphingomyelinase, which inhibits the production of TDEs and exerts an antitumor effect. DC-derived exosomes contain tumor antigens; therefore, TAE-DC vaccines have been developed to activate T cells in the TME 307. Macrophage-derived exosomes can also be used to reverse the immunosuppressive effect of M2 macrophages and induce polarization of macrophages to the M1 phenotype 308. In addition, artificially developed antigen-capturing liposomes can capture and transport tumor antigens to DC, subsequently enhancing antigen cross-presentation 309.

### Tumor biomarkers related to lipid metabolism

Several key enzymes and products of lipid metabolic pathways can be used as markers for the development of tumors and tumor immunity 310. The function, characteristics, and expression of lipid metabolism-related markers in different types and stages of tumors are summarized in Table 1. Notably, an enhanced expression of cholesterol-related genes is associated with low immunotherapy response, which indicates their potential value as immunotherapy biomarkers 87.

## Conclusions

Lipid metabolic reprogramming plays an essential role in TME and cancer immunotherapy. Here, we described the characteristics, mechanism, and role of lipid metabolic reprogramming in tumor and immune cells interacting in the TME. Importantly, lipid metabolic reprogramming even could determine cell fate, which is manifested in the regulation of different cell death modes, especially ferroptosis. Further, we summarized the potential clinical applications of targeting lipid metabolic reprogramming for antitumor therapies and as a tumor biomarker. Given the complexity of lipid metabolic reprogramming and the continuous research efforts to identify the critical genes and pathways involved, researchers will discover several candidate genes/proteins for diagnostic and therapeutic purposes. In addition, lipid metabolic biomarkers have shown potential for monitoring immunotherapy; therefore, they should be the primary focus of future research. Overall, an improved understanding of lipid metabolic reprogramming will provide useful insights into mechanisms of tumorigenicity and may lead to the development of novel therapeutic approaches for patients with cancer.

## Figures and Tables

**Table 1:** Key enzymes of lipid metabolism and their expression in tumors.

Category	Stage	Key enzyme	Function	Effect	Expression
FA	Uptake	CD36	Absorb LCFA, TC and oxLDL	FAO, angiogenesis, EMT, lipid peroxidation, and chemoradiotherapy resistance	Highly expressed, positively correlated Tumor and immunity marker
		FATP	Absorb and transport LCFA		
		FABPpm/FABP	Absorb and transport FA		
	Synthesis	ACLY	Catalyze AcCoA to citric acid	Glucose, glutamine, lipid metabolism, Wnt/β-catenin signaling, histone acetylation, and anti-tumor immunity	
		ACSS2	Catalyze acetate to AcCoA	Compensate for ACLY, lysosomal synthesis and autophagy	Highly expressed except CRC and gastric cancer, positively correlated Tumor and immunity marker
		ACC1/2	Catalyze AcCoA to malonyl-CoA	Balance between FA synthesis and decomposition	Highly expressed except lung adenocarcinoma Tumor marker
		FASN	Catalyze malonyl-CoA to palmitic acid	Glycolysis, amino acid metabolism, and ferroptosis	Highly expressed, positively correlated Tumor marker
	Desaturation	SCD	Catalyze palmitic acid to palmitic acid, stearic acid to oleic acid	Ratio of MUFA/SFA in membrane, tumor proliferation, apoptosis, inflammation, invasion, and metastasis	High expressed, positively correlated with ccRCC but negatively with breast cancer and liver cancer Tumor marke
		FADS2	Catalyze LA and ALA to PUFA, palmitic acid to sapienate	Compensate for SCD, ratio of SFA/UFA and ω-3/ω-6PUFA in membrane	Highly expressed in SCD independent or suppressed tumors
	Elongation	ELOVL2/5/6	Catalyze palmitic acid to stearic acid, palmitic acid to oleic acid, sapienate to coctadecenoate, LA and ALA to PUFA		Highly expressed in low malignancy but lowly expressed in high malignancy Tumor marke
	Storage	ACSL	Catalyze FA and AcCoA to FA-CoA	TAG synthesis, FAO, autophagy, Lands cycle, and ferroptosis	ACSL3 highly expressed in AR+ tumors but lowly expressed in ER-ones ACSL4 highly expressed in ER- tumors but lowly expressed in ER+ and AR+ ones
		ACBP	Transport acyl-CoA		High expressed
		GPAT	Catalyze glycerol 3-phosphate and acyl-CoA to LPA		Highly expressed in active TAG synthesis tumors, negatively correlated except LIPIN
		AGPAT	Catalyze LPA and acyl-CoA to PA		
		PAP (LIPIN)	Catalyze PA to DAG	Regulation of lipid metabolism	
		DAG	Catalyze DAG and acyl-CoA to TAG		
	Lipolysis	ATGL	Catalyze TAG to DAG and FA	Cancer-associated cachexia	Highly expressed except lung cancer and head and neck squamous cell carcinoma
		HSL	Catalyze DAG to MG and FA		Highly expressed in high malignancy
		MAGL	Catalyze MG to Gly and FA	Cancer stem cells marker	
	Decomposition	ACSL/ACBP	Ditto		Highly expressed Tumor and immunity marker
		CAT1	Transport LCFA-CoA into mitochondria		
		β-oxidase system	Catalyze FA-CoA to AcCoA, NADH and FADH2		
TC	Uptake	LDLR	Absorb TC	EGFR, PI3K/Akt and SREBP1 signaling	Highly expressed, positively correlated Immunity marker
	Synthesis	HMGCR	Catalyze HMG-CoA to MVA		Lowly expressed, negatively correlated in lipid-rich tumors but highly expressed in other solid and hematological tumors Immunity marker
		SQS	Catalyze FPP to squalene		Highly expressed especially tumors with low HMGCR, negatively correlated Immunity marker
		SQLE	Catalyze squalene to 2,3-epoxy squalene		
	Storage	ACAT1	Catalyze TG to CE	TC uptake, synthesis, storage, flow to membrane, and tumor immunity	Highly expressed, positively correlated Tumor and immunity marker
	Efflux	ABCA1/G1	Transport TC to ApoA1 and HDL-C	Tumor suppressor gene, tumor survival and EMT	Lowly expressed in p53-mutated tumors, highly expressed in lipid-rich tumors often with low HMGCR Tumor marker (including serum HDL-C and HDLR)
	Decomposition	CYP27A1	Catalyze TC to 27-HC		Highly expressed in endothelial cells and macrophages
Ketone body	Synthesis	HMGCL	Catalyze HMG-CoA to AcAc		Highly expressed in melanoma and GBM
		β-hydroxybutyrate dehydrogenase	Catalyze AcAc to BHB		
	Decomposition	BDH1	Catalyze BHB to AcAc		Highly expressed in "ketone metabolic subtype" tumors
		OXCT1	Catalyze AcAc to AcAcCoA and succinic acid		
		ACAT1	Catalyze AcAcCoA to AcCoA		
PGs	Synthesis	COX-1/2	Catalyze AA to PGG2 and PGH2	Chronic inflammation into tumors, apoptosis, proliferation, invasion, metastasis, angiogenesis, immunosuppression and drug resistance	Highly expressed in solid and hematological tumors, especially Inflammation-associated ones except SCL
		LOX	Produce LTs		
		TXA2 synthetase	Catalyze PGG2 and PGH2 to TXA2		
		PGES	Catalyze PGG2 and PGH2 to PGE2		
	Decomposition	15-PGDH	Decompose PGE2 Antagonize COX-2		Lowly expressed
GPLs remodeling	Lands cycle	PLA_2_	Participate in the deacylation of GPL		Vary with tumor types and stages, and subtypes of enzymes Tumor marker
		LPCAT	Participate in the reacylation of GPL		
Ferroptosis	Peroxidation	ACSL4 and LPCAT3	Ditto		
		LOX	Participate in the generation of PLOOH		
	Antioxidation	SXC	Transport cysteine intracellularly and pump glutamate out		Highly expressed
		GPX4	Scavenge PLOOH by catalyzing GSH to GSSG		
		FSP1	Catalyze CoQ10 to ubiquinol		
	Transport	TFRC	Combine with TF and absorb Fe3+		Highly expressed
		FPN1	Pump excessive Fe2+ extracellularly		

**Table 2:** Metabolic reprogramming in tumor-associated immune cells.

Cell type			Functions	Reprogramming
Anti-tumor immune cells	Adaptive immunity	Teff cells	Antigen-specific cytotoxic effects, perforin, granzyme, γ-IFN, TNF-α, and FasL	Enhance glycolysis, TCA cycle, OXPHOS, and PPP, use lactic acids Enhance FA synthesis, FAO, and TC uptake, use ketone bodies
		CD4+ conv cells	γ-IFN and TNF-α Activate Teff cells, NK cells, and B cells	
		CD8+T mem cells	Long-term tumor immunity	Enhance OXPHOS Enhance FAO, CD36-mediated FA uptake, acetate-mediated FA synthesis
		B cells	Tumor-specific antibodies, ADCC effect, CDC effect, granzyme B, and TRAIL Activate T cells	Increase oxygen consumption Enhance glycolysis and PPP, upregulate GLUT
	Innate immunity	NKT cells	γ-IFN, perforin, FasL and TRAIL Activate NK cells, T cells, and DCs	Depend on FA and TC
		NK cells	ADCC effect, perforin, granzyme, γ-IFN, TNF-α, FasL, and TRAIL	Enhance glycolysis and OXPHOS, inhibit gluconeogenesis Upregulate LDLR
		M1 macrophages	ADCC effect, TNF-α, IL-1β, COX-2, iNOS, ROS, NO, and antigen presentation	Enhance glycolysis and PPP Tend to PUFAs
		DCs	Antigen presentation, recognize DAMP, and PAMP Activate NK cells, NKT cells, and Teff cells	Enhance glycolysis and inhibit OXPHOS Tend to PL and TAG (especially PUFA) but TC and CE
		N1 neutrophils	Phagocytosis, ROS, RNS, and α/β-IFN Activate NK cells and inhibit IL-17 T cells	Enhance glycolysis, PPP, and glycogen metabolism
Pro-tumor immune cells	Adaptive immunity	Treg cells	IL-4, IL-10, TGF-β, adenosine, CTLA-4, PD-1, and LAG-3 Inhibit APCs and T cells	Enhance OXPHOS, FAO, FA and TC synthesis, upregulate CD36 and FANS Downregulate GLUT1 and upregulate MCT1, use lactic acids
		Breg cells	IL-10, TGF-β, ROS, PD-L1 and CTLA-4 Inhibit T cells and NK cell, and activate Treg cells, MDSCs and M2 macrophages	Abnormal TC metabolism Use lactic acid, enhance glycolysis
	Innate immunity	M2 macrophages	IL-10, TGF-β, VEGF, EGFR, and MMPs	Enhance FA synthesis, FAO, and OXPHOS, upregulate CD36 and ABCG1
		MDSCs	IL-10, TGF-β, Arg1, NO, and ROS Inhibit T cells and activate Treg cells	Enhance FAO, OXPHOS, upregulate CD36, LDLR and FATP2 Enhanced glycolysis
		N2 neutrophils	ROS, RNS, Arg1, MPO, NE, and NETs Inhibit T cells and NK cells	Enhance lipid uptake, LD formation, and inhibit FAO and LD degradation Upregulate GLUT1

**Table 3:** Therapies targeting lipid metabolism to sensitize immunotherapy.

Target		Drug	Phase	Tumor type	Trial number	Effect
FA	Reduce SFA and TC in blood	PCSK9 inhibitor, statins, fibrates, and ezetimibe	Preclinical, phase I and II	Melanoma, Colon cancer, and breast cancer	NA	Promote Teff cells and inhibit Treg cells
	Increase ω-3PUFA in blood	Diet therapy	Phase II and III	Head and Neck Cancer	NCT05101889	Inhibit PGs and NF-kB pathway, regulate autophagy
	Inhibit CD36	CD36 antibody, siRNA, siRNA, and CD36 inhibitor	Preclinical	Melanoma, colon cancer, and lymphoma	NA	Promote DCs and Teff cells, inhibit Treg cells, and induce ferroptosis
	Inhibit FATP	FATP inhibitor: Lipofermata and (Z)-Ligustilide	Preclinical	NSCLC, colon cancer, and lymphoma	NA	Inhibit MDSCs
	Inhibit ACLY	ACLY inhibitor: LY294002, SB-204990, and NDI-091143	Preclinical	GBM	NA	Affect Treg cells, T cells
	Inhibit ACSS2	ACSS2 inhibitor: VY-3-135	Preclinical	CESC and TNBC	NA	Affect PD-L1, B cells, T cells, and CAFs
	Inhibit ACSL4	ACSL4 inhibitor: troglitazone, and PRGL493	Preclinical	Liposarcoma	NA	Inhibit ferroptosis of Teff cells
	Inhibit ACC	ACC inhibitor: PF-05175157, Firsocostat, and CP-640186	Phase II	Esophageal cancer	ChiCTR- ICR-15005940	Promote T cells and inhibit M2 macrophages
	Inhibit FASN	FASN inhibitor: omeprazole, C75, orlistat, and UCM05	Phase II	TNBC, thyroid cancer, neuroblastoma	NA	Induce ferroptosis, inhibit M2 macrophages, and Treg cells
	Inhibit SCD1	SCD1 inhibitor: BZ36, A939572, MK-8245, and XEN723	Phase II	Breast cancer	NA	Induce ferroptosis, endoplasmic reticulum stress, mitochondrial apoptosis, and autophagy
	Inhibit FADS	FADS inhibitor: SC-26196	Preclinical	Colon cancer	NA	Reduce ω-3/ω-6PUFA and induce ferroptosis
	Inhibit MAGL	MAGL inhibitor: JZL184, JJKK048, and JW 642	Preclinical	Multiple tumors	NA	Increase LPA, LPC, and PGE2
	Inhibit CPT1	CPT1 inhibitor: etomoxir, McN3716, teglicar, and perhexiline	Preclinical	Breast cancer, colon cancer, lung cancer, and prostate cancer	NA	Inhibit TAMs and MDSCs, and enhance T cells
PGs	Inhibit COX /PGES	COX1/2 inhibitor: NSAIDs mPGES1 inhibitor	Preclinical and phase II	Melanoma, CRC, prostate cancer, and GBM	NA	Affect IDO1
TC	Inhibit HMGCR	HMGCR inhibitor: statins and HMG499	Phase II	Breast cancer, leukemia, liver cancer, and myeloma	NA	Reduce ROS and PD-L1, enhance T cells, and inhibit ferroptosis
	Inhibit SQS	SQS inhibitor: zalagozic acid A	Preclinical	Liver cancer	NA	Increase TNF-α, NF-κB, and MMP1
	Inhibit SQLE	SQLE inhibitor	Preclinical	PAAD	NA	Affect immune cell and immune checkpoint
	Inhibit ACAT1	ACAT1 inhibitor: avamaib	Preclinical	Melanoma and cholangiocarcinoma	NA	Enhance Teff cells
Regulation	Activate AMPK	AMPK agonist: metformin, acardixin (AICAR)	Phase III	Breast cancer	NCT01101438	Reduce PD-1
	Inhibit mTOR	mTOR inhibitor: rapamycin analogue and everolimus	Phase III	AML and breast cancer	ChiCTR-OPC-14005488	Inhibit Treg cells, Teff cells, and NK cells
	Activate PPARγ	PPARγagonist: bezafibrate and troglitazone	Preclinical, phase I, and II	Thyroid tumor and breast cancer	NA	Enhance Teff cells
	Inhibit PPARα	PPARαinhibitor: adriamycin and chophylline	Preclinical	Breast cancer, liver cancer, and colorectal cancer	NA	Enhance DCs
	Inhibit SREBP1	SREBP1 inhibitor: siRNA, pseudoprotodioscin, and fatostatin	Preclinical	CRC and TNBC	NA	Inhibit Treg cells and M2 macrophages
	Activate LXR	LXR agonist: RGX-104	Preclinical and phase I	Breast cancer	NCT02922764	Inhibit MDSCs and enhance M1 macrophages
Ketone body	Increase BHB	KD therapy	Phase II and I	Breast cancer, ovarian cancer, endometrial cancer, and breast cancer	NCT03795493	Enhance anti-oxidation of immune cells, regulate p53 and mTOR pathways, and control inflammation
		Supplement BHB				
Inhibit autophagy		Autophagy inhibitor: hydroxychloroquine (HCQ) and autophinib	Phase II	CRC, pancreatic cancer, and melanoma	NCT02316340	Increase tumor immunogenicity and enhance T cells
Ferroptosis	Inhibit SLC7A11	Sorafenib	Clinal	AML, HCC, neuroblastoma, NSCLC, and RCC	NCT03247088, NCT02559778, and NCT00064350	Inhibit ferroptosis-related antioxidant system
		Sulfasalazine	Phase II	Breast cancer and GBM	NCT04205357, NCT01577966, and NCT03847311	
	Inhibit GPX4	Altretamine	Clinical	Lymphoma and sarcoma	NCT00002936	
		Withaferin A	Phase II	Breast cancer, osteosarcoma, and HCC	NCT04092647 and NCT00689195	
	Inhibit GCL	Buthionine sulfoxide	Phase I	Melanoma and neuroblastoma	NCT00002730, NCT00005835, and NCT00661336	
	Activate iron metabolism	Neratinib	Clinical	Breast cancer and CRC	NCT04366713, NCT03377387, and NCT03457896	Enhance ferroptosis-related peroxidation system
		Salinomycin	Clinical	Multiple cancer	NA	
		Lapatinib	Clinical	Breast cancer	NCT03085368, NCT00356811, and NCT00667251	
Drug carriers	Exosome	TAE-DC	Preclinical	Pancreatic cancer	NA	Enhance T cells
		Exosome-derived miRNA	Preclinical	Breast cancer	NA	Inhibit M2 macrophages and induce M1 macrophages
	Liposome	Antigen-capture spike liposomes	Preclinical	Multiple cancer	NA	Capture and transport tumor antigens to DCs

## Abbreviations

27-HC: 27-hydroxycholesterol
4EBP: EIF4E binding protein
5-HPETE: 5-hydroxyperoxyeicosatetraenoic acid
AAD: Anti-angiogenic drug
ABCA1: ATP-binding cassette transporter A1
ABCG1: ATP-binding cassette transporter G1
ACAT: Cholesterol acyltransferase/acetoacetyl-CoA sulfhydrylase
ACC/ACACA: Acetyl-CoA carboxylase
ACLY: ATP-citrate lyase
ACSL: Acyl-CoA synthase long-chain family
ACSS: Acetyl-CoA synthase
AdA: Adrenocorticotropic acid
ADCC: Antibody dependent cell mediated cytotoxicity
AGPAT: acylglycerol-3-phosphate acyltransferase
ALOX: Arachidonic acid lipoxygenase
AML: Angiomyolipoma
AMPK: AMP-activated protein kinase
APCs: Antigen-presenting cells
AR: Androgen receptor
ATGL: Adipose triglyceride lipase
AcAc: Acetoacetic acid
AcAcCoA: Acetoacetyl-CoA
AcCoA: Acetyl coenzyme A
Apo: Apolipoprotein
Arg1: Arginase 1
BCR: B-cell receptor
BDH1: 3-hydroxybutyrate dehydrogenase
BHB: β-hydroxybutyrate
Breg: Regulatory B
CAFs: Cancer-associated fibroblasts
CALR: Calreticulin
CD4+conv: Antitumor CD4+
CDC: Complement dependent cytotoxicity
CDK: Cell cycle protein-dependent kinase
CE: Cholesterol ester
CESC: Cervical squamous cell carcinoma
CL: Cardiolipin
COX: Cyclo-oxyganese
CPT1: Carnitine lipid acyltransferase 1
CRC: Colorectal cancer
CoQ10: Coenzyme Q10
Cys: Cysteine
DAG: Diacylglycerol
DAMP: Damage-associated molecular pattern
DC: Dendritic cells
DGAT: Diacylglycerol acyltransferase
DHA: Docosahexaenoic acid
DLAT: Dihydrolipoyl Transacetylase
Drp1: Hairpin-associated protein 1
ECM: Extracellular matrix
EGFR: Epidermal growth factor receptor
ELOVL: Elongation of very long chain fatty acid
EMT: Epithelial-mesenchymal transition
ER: Estrogen receptor
ETC: Electron transport chain
Eps: PGE receptor
FA: Fatty acid
FABPpm: Plasma membrane fatty acid binding protein
FACoA: Acyl-CoA
FADS: Fatty acid desaturase
FAO: Fatty acid oxidation
FASN: Fatty acid synthase
FATP: Fatty acid transport protein
FBP: Fructose-1,6-bisphosphatase
FDPS: Farnesyl diphosphate synthase
FDX1: Iron oxygen reducing protein 1
FFA: Free fatty acid
FGF2: Fibroblast growth factor 2
Foxp3: Forkhead box protein 3
FPN: Iron transport protein
FPP: 15-carbon pyrophosphate farnesylate
FSP1: Iron action suppressor protein 1
G3P: 3-phosphoglycerol
G6P: Glucose-6-phosphatase
GBM: Glioblastoma
GCL: Glutamate cysteine ligase
GGPP: Geranyl geranyl pyrophosphate
GK: Glycerol kinase
GLUT: Glucose transporter protein
Gly: Glycerin
GM-CSF: Granulocyte-macrophage colony-stimulating factor
GPAT: Glycerol-3-phosphate acyltransferase
GPCR: G protein-coupled receptor
GPLs: Glycerol phospholipids
GPX4: Glutathione peroxidase 4
GSH: Reduced glutathione
GSSG: Oxidized glutathione
Glu: Glutamate
HCC: Hepatocellular carcinoma
HDAC: Histone deacetylase
HDL: High density lipoproteins
HFD: High-fat diet
HIF-1α: Hypoxia-inducible factor-1α
HISLA: HIF-1α stable lncRNA
HK: Hexokinase
HMGB1: High mobility group protein 1
HMGCR: HMGCoA reductase
HMGCS1: HMGCoA synthase 1
HMGCoA: 3-hydroxy-3-methylglutaryl-CoA
HNE: 4-hydroxynonenoic acid
HSL: Hormone-sensitive lipase
HSP: Heat shock protein
ICD: Immunogenic cell death
ICIs: Immune checkpoint inhibitors
IDH1: Isocitrate dehydrogenase 1
IDO1: Indoleamine 2,3-dioxygenase 1
IGFR: Insulin-like growth factor receptor
iNOS: Inducible nitric oxide synthase
INSIG: Insulin-inducible gene protein
IP3: Inositol 1,4,5-triphosphate
IPP: Isopentenyl pyrophosphate
lysoPLs: Lysophospholipids
KD: Ketogenic diet
Kbhb: β-hydroxybutyrylation
Kbu: Butyrylation
Kcr: Crotonylation
Kla: Lactonization
Kma: Malonylation
Kpr: Malonylation
Ksucc: Succinylation
LA: Linoleic acid
LAL: Lysosomal acid lipase
LAR: Luminal androgen receptor
LCFA: Long chain fatty acids
LD: Lipid droplet
LDLR: Low density lipoprotein receptor
LIP: Unstable iron pool
LKB1: Liver kinase B1
LLO: Lipid-linked oligosaccharide
LOX: Lipoxygenase
LPIN1/PAP: Phosphatidate phosphatase
LPA: Lysophosphatidic acid
LPC: Lysophosphatidylcholine
LPCAT: Lysophosphatidylcholine acyltransferase
LPO: Lipid peroxidation
LTs: Leukotrienes
LXRE: LXR response element
LXRs: Liver X receptors
MAGL: Monoacylglycerol lipase
MCT1: Monocarboxylate transporter 1
MDA: Malondialdehyde
MDSCs: Myeloid suppressor cells
MG: Monoglyceride
MHC I: Major histocompatibility complex 1
MPO: Myeloperoxidase
mTOR: Mammalian target of rapamycin protein
MUFA: Monounsaturated fatty acids
MVA: Mevalonate
MVD: Mevalonate diphosphate decarboxylase
MVK: Mevalonate kinase
NE: Neutrophil elastase
NETs: Neutrophil extracellular trap network
NK: Natural killer
NLRP3: Nod-like receptor protein 3
NSAIDs: Non-steroidal anti-inflammatory drugs
NSCLC: Non-small cell lung cancer
OGR1: G protein-coupled receptor 1
OXCT1: 3-succinyl coenzyme A transferase
oxLDL: Oxidized low-density lipoprotein
OXPHOS: Oxidative phosphorylation
PA: Phosphatidic acid
PAAD: Pancreatic adenocarcinoma
PARP: Polyadenosine diphosphate ribose polymerase
PC: Phosphatidylcholine
PCK1: Phosphoenolpyruvate carboxykinase
PDH: Pyruvate dehydrogenase
PE: Phosphatidylethanolamine
PGES: Prostaglandin E2 synthase
PGs: Prostaglandins
PI: Phosphatidylinositol
PI3K: Phosphoinositide 3-kinase
PIM1: Serine/threonine kinase
PIP2: Phosphati-dylinositol-4,5-bisphosphate
PIP3: Phosphatidylinositol-3,4,5-trisphosphate
PKC: Protein kinase C
PKM2: Pyruvate kinase M2
PL: Phospholipids
PLA2: Phospholipase A2
PLC: Phospholipase C
PLOOH: Phospholipid peroxide
PMVK: Mevalonate phosphate kinase
POR: P450 oxidoreductase
PPARs: Peroxisome proliferator-activated receptors
PPP: Pentose phosphate pathway
PPRE: PPAR response element
PS: Phosphatidylserine
PUFA: Polyunsaturated fatty acid
RCC: Renal cell carcinoma
RCD: Regulatory cell death
RNS: Reactive nitrogen
ROS: Reactive oxygen species
RTA: Free radical trapping antioxidant
RTK: Receptor tyrosine kinase
RXR: Retinol-like X receptor
S6K: Ribosomal protein S6 kinase
SCAP: SREBP cleavage-activating protein
SCD: Stearoyl-CoA desaturase
SCF: Stem cell factor
sec-tRNA: Selenocysteine-tRNA
SGK: Serum and glucocorticoid induced kinase
SOD: Superoxide dismutase
SQLE: Squalene epoxidase
SQS: Squalene synthase
SRE: Sterol regulatory element
SREBPs: Sterol regulatory element binding proteins
SXC: System x-
TAG: Triglycerides
TAMs: Tumor-associated macrophages
TANs: Tumor-associated neutrophils
TC: Total cholesterol
TCA: Tricarboxylic acid
TCR: T-cell antigen receptor
TDE: Tumor-derived exosomes
TFRC: Transferrin receptor
TGF-β: Transforming growth factor-β
TLR: Toll-like receptor
TME: Tumor microenvironment
tmTNF-α: Transmembrane tumor necrosis factor alpha
TNBC: Triple-negative breast cancer
TRAIL: Tumor necrosis factor-associated apoptosis-inducing ligand
TXs: Thromboxane
TZDs: Thiazolidinedione
Teff: Effector CD8+ T
Tmem: Memory CD8+ T
Treg: Regulatory T
UFA: Saturated fatty acids
VEGF: Vascular endothelial growth factor
XO: Xanthine oxidase
α-KG: α-ketoglutarate
γ-IFN: γ-interferon
